# Targeting DNA Methyltranferases in Urological Tumors

**DOI:** 10.3389/fphar.2018.00366

**Published:** 2018-04-13

**Authors:** Ângela Marques-Magalhães, Inês Graça, Rui Henrique, Carmen Jerónimo

**Affiliations:** ^1^Cancer Biology and Epigenetics Group – Research Center, Portuguese Oncology Institute of Porto, Porto, Portugal; ^2^Department of Pathology, Portuguese Oncology Institute of Porto, Porto, Portugal; ^3^Department of Pathology and Molecular Immunology, Institute of Biomedical Sciences Abel Salazar, University of Porto, Porto, Portugal

**Keywords:** bladder cancer, kidney cancer, prostate cancer, testicular cancer, DNA methylgtransferases inhibitors, pre-clinical studies, clinical trials

## Abstract

Urological cancers are a heterogeneous group of malignancies accounting for a considerable proportion of cancer-related morbidity and mortality worldwide. Aberrant epigenetic traits, especially altered DNA methylation patterns constitute a hallmark of these tumors. Nonetheless, these alterations are reversible, and several efforts have been carried out to design and test several epigenetic compounds that might reprogram tumor cell phenotype back to a normal state. Indeed, several DNMT inhibitors are currently under evaluation for therapeutic efficacy in clinical trials. This review highlights the critical role of DNA methylation in urological cancers and summarizes the available data on pre-clinical assays and clinical trials with DNMT inhibitors in bladder, kidney, prostate, and testicular germ cell cancers.

## Introduction

Urological cancers, comprising those primarily originating in bladder, kidney, prostate and testis, are an heterogeneous class of malignancies accounting for significant morbidity and mortality worldwide (Ferlay et al., 2010). Prostate, bladder and renal cancers are among the 10 most frequent malignancies in men, whilst testicular germ cell tumors (TGCT) are less prevalent, although they represent the most common cancer in young men (Torre et al., 2015). In 2012, there was an estimated 14.1 million new cases diagnosed and 8.2 million deaths due to urological cancers, worldwide (Torre et al., 2015). Importantly, the majority of these tumors are asymptomatic at the earliest disease stages, at which the curability rate is higher, and there are only a few body fluid-based biomarkers proposed for early disease detection (Jerónimo and Henrique, 2014; Ellinger et al., 2015). Due to the high incidence and mortality rates, it is imperative to improve not only early detection approaches but also to develop new therapeutic strategies.

Both genetic and epigenetic features contribute to the definition of cellular identity in human organs and tissues by establishing an appropriate organism physiology (Kelly and Issa, 2017). Epigenetics may be defined as the study of heritable modifications of DNA or associated proteins which carries information related to gene expression during cell division. Unlike genetic abnormalities, epigenetic changes do not alter the DNA sequence and are potentially reversible (Baylin and Jones, 2016). In mammals, epigenetic inheritance is important for pre-implantation and fetal development, as well as cell and tissue differentiation (Meissner, 2010; Skinner, 2011; Saitou et al., 2012). Epigenetic regulation comprises four major mechanisms: DNA methylation, histone post-translational modifications or chromatin remodeling, histone variants, and non-coding RNAs' regulation (Kelly and Issa, 2017). All cancer types harbor several epigenetic aberrations that correlate with malignant transformation and tumor progression (Esteller, 2008). Due to the reversibility of these alterations, modulation of the epigenetic machinery could provide new attractive therapeutic approaches for cancer (Azad et al., 2013).

This review summarizes the recent advances of epigenetic therapy, namely DNA methylation inhibitors (DNMTi), for treatment of urological cancers.

## DNA methylation

DNA methylation is one of the best studied epigenetic mechanisms in eukaryotes, consisting of the covalent addition of a methyl group to the 5′ carbon of a cytosine ring, mainly within a CpG dinucleotide, yielding a new DNA base, 5-methylcytosine (5 mC). This process is catalyzed by DNA methyltransferases (DNMTs) using S-adenosyl-L-methionine (SAM) as the methyl group donor (Goldberg et al., 2007; Lopez-Serra and Esteller, 2008). There are three well known DNMTs: DNMT1, DNMT3A, and DNMT3B. Whilst DNMT1 is mainly responsible for the maintenance of parental cell DNA methylation within the newly synthesized DNA strand during cell division (Leonhardt et al., 1992; Robert et al., 2003; Sharif et al., 2007), both DNMT3A and DNMT3B have *de novo* methylation activity (Okano et al., 1998; Chen et al., 2003). Importantly, the addition of the methyl group to cytosine does not interfere with the Watson-Crick base paring of the nucleotide. This group is inserted in the major groove of DNA, where it may be efficiently recognized by DNA-interacting proteins (Jurkowska et al., 2011).

**Figure 1:**
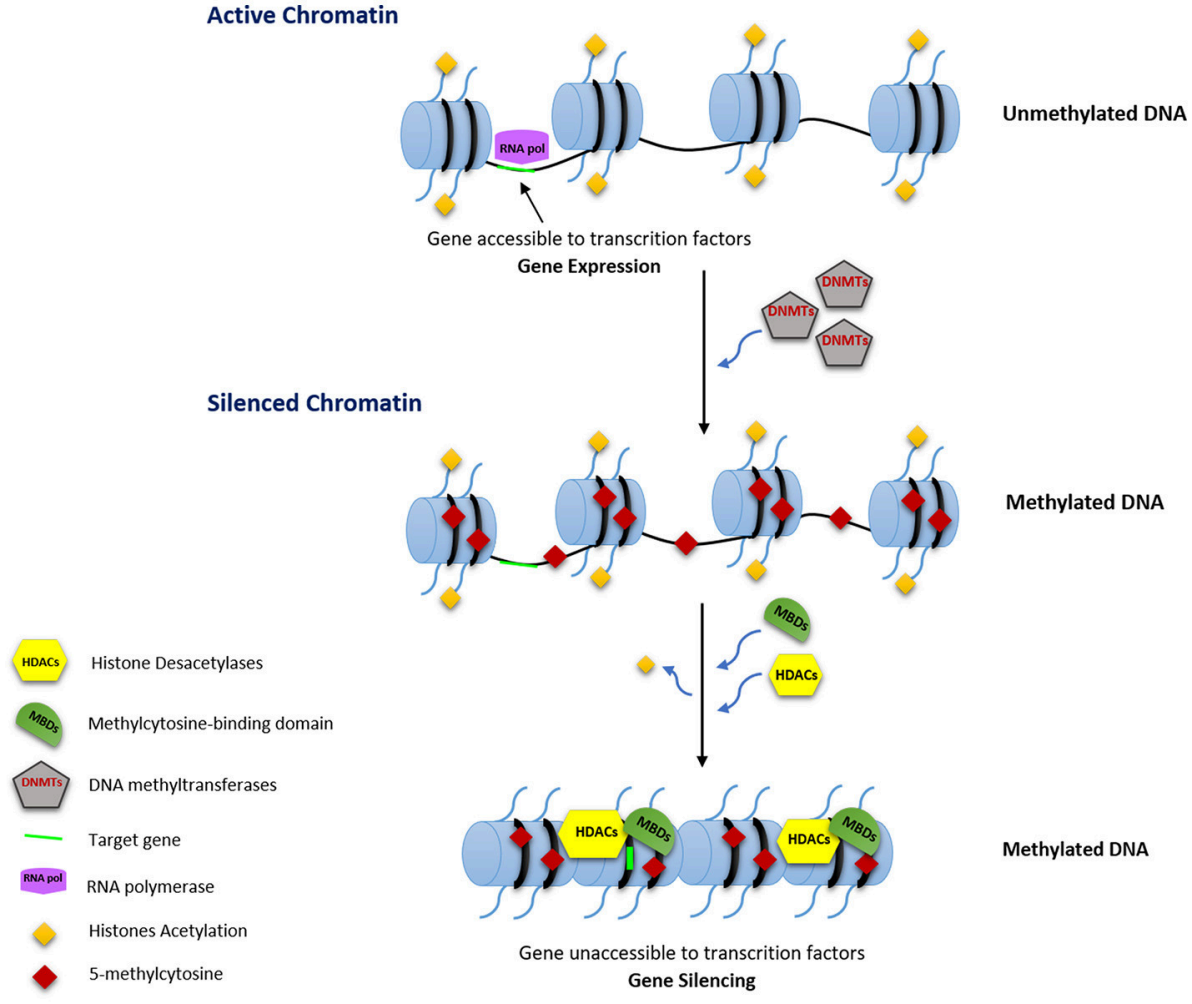
Transcription regulation by DNA methylation. RNA pol, RNA polymerase; DNMTs, DNA methyltransferases; HDACs, Histone Deacetylases; MBDs, Methylcytosine-binding domain.

DNA methylation is closely linked to control of gene expression either by inhibiting the binding of transcription factors through direct methylation of CpG dinucleotides within their binding sites and/or by acting as binding sites for methyl-CpG binding proteins (MBPs). MBPs, associated with other factors such as histone deacetylases (HDACs), can establish repressive chromatin structures (Figure 1; Jones et al., 1998; Robertson and Wolffe, 2000; Klose and Bird, 2006).

Methylation patterns are clonally inherited and preserved in daughter cells through replicative DNA methylation accomplished by DNMT enzymes (Stein et al., 1982). DNA demethylation may occur through an active or passive mechanism, or through a combination of both (Seisenberger et al., 2013; Guo et al., 2014). Pioneering studies demonstrated the occurrence of a global and active loss of methylation of the paternal genome during embryogenesis. Contrarily, maternal genome is passively demethylated due to DNA replication during the subsequent cell divisions (Mayer et al., 2000; Santos et al., 2002; Guo et al., 2014). Active demethylation includes oxidation of 5 mC to 5-hydroximetylcytosine (5 hmC) mediated by TET (ten-eleven translocation) proteins, and subsequent targeting by BER (Base Excision Repair) pathway (Seisenberger et al., 2013), whereas passive demethylation consists in gradual loss of methylation in the early embryo through lack of maintenance during DNA replication, such as predominant exclusion of DNMT1 (Howell et al., 2001).

Changes in DNA methylation patterns have been described in several human diseases, including cancer (Robertson and Wolffe, 2000). In fact, gain in DNA methylation at actively transcribed gene promoters, normally unmethylated, may lead to a selective inactivation of genes, including tumor suppressor genes (TSGs), in cancer. Concomitantly, DNA demethylation of normally methylated regions, such as repetitive sequences (satellite DNA and transposable elements) which account for the bulk of CpG methylation in the genome, have been associated with chromosomal instability and activation of proto-oncogenes (Esteller, 2008; Mohanty et al., 2016). Importantly, tumor-specific promoter hypermethylation often occurs in the midst of widespread DNA hypomethylation (Baylin and Jones, 2016). Until now, the mechanisms underlying these aberrant DNA methylation patterns remain largely unknown. Nevertheless, some studies have suggested that these modifications possibly arise early in tumor development, depending on the cancer type (Coolen et al., 2010; Joyce et al., 2016).

## DNMTs inhibitors

**Figure 2:**
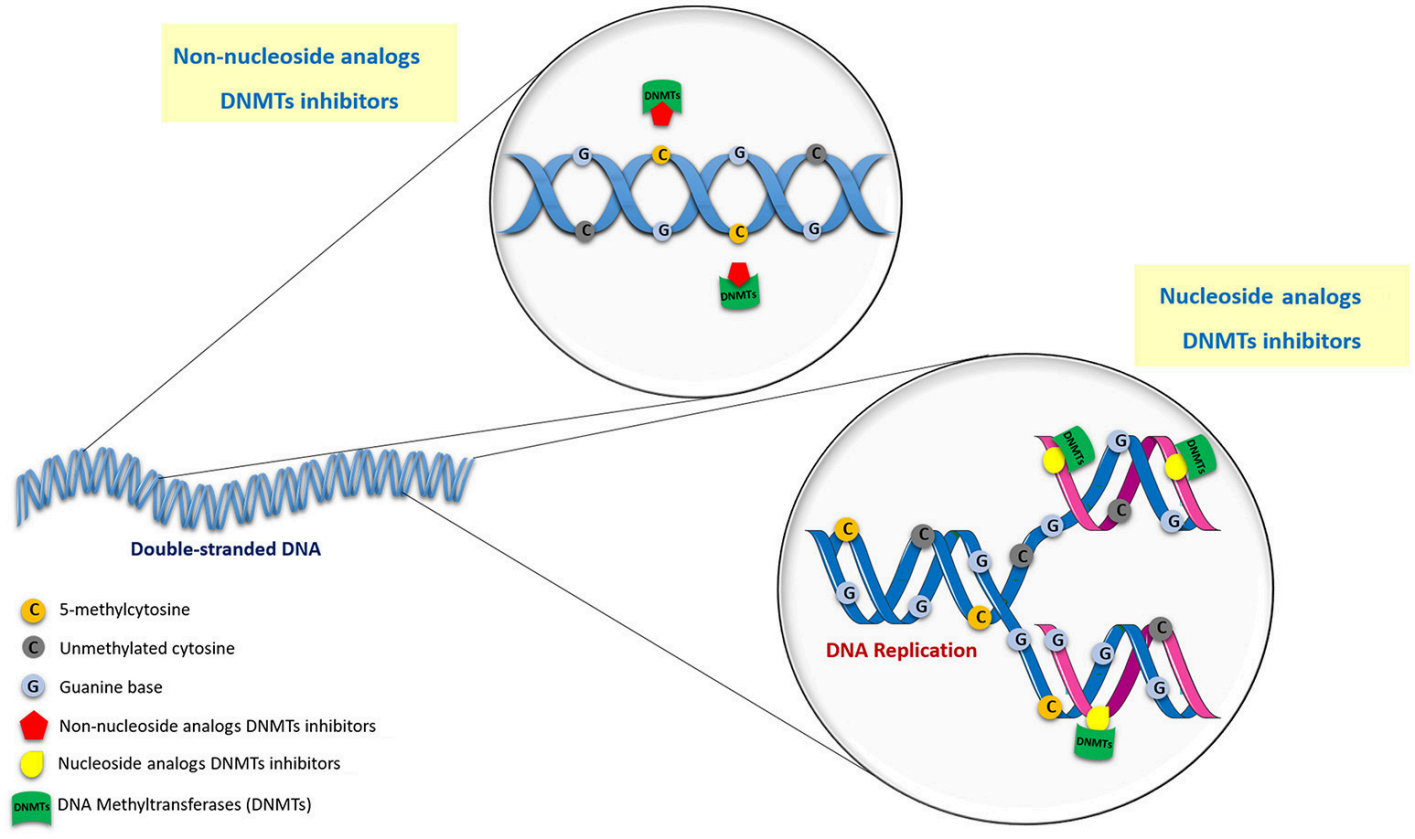
Mechanism of action of nucleoside and non-nucleoside DNMT inhibitors.

Over the last decade, several compounds were found to erase abnormal methylation patterns by irreversibly inhibiting the enzymatic activity of DNMTs and triggering their proteosomal degradation (Kelly et al., 2010; O'rourke et al., 2013). This, in turn, actively contributes to neoplastic cell phenotype attenuation by inducing cell differentiation and tumor cell death, leading to significant clinical benefits (Dhanak and Jackson, 2014). Indeed, two epigenetic compounds that target DNA methylation have already been approved by Food and Drug Administration (FDA) and European Medicines Agency (EMA) for cancer treatment. Generically, DNMTi may be divided into two main classes depending on their mode of action: nucleoside and non-nucleoside analogs (Figure 2; Erdmann et al., 2014).

### Nucleoside analogs

Nucleoside analogs comprise a modified cytosine ring which is connected to either a ribose or deoxyribose moiety and may, be integrated into DNA or RNA, replacing cytosines. When incorporated into DNA, during S phase of the cell cycle, they covalently bind and inhibit DNMTs on the DNA strand, inducing DNA damage and cell death (Goffin and Eisenhauer, 2002; Issa and Kantarjian, 2009). Therefore, these compounds can deplete DNMTs, resulting in a global loss of cytosine methylation patterns in daughter cells genome after successive DNA replications. This process might be responsible for the re-expression of abnormally silenced growth regulatory genes leading to chromatin extension, cell cycle arrest, and induction of cellular differentiation (Stresemann and Lyko, 2008; Issa and Kantarjian, 2009). Unfortunately, the exact mechanism of action of these compounds remains unclear. It was proposed that the cytotoxic effect of 5-aza-2′-deoxycytidine is directly linked to its covalent binding to DNMTs in DNA strand, being expected that tumor cells with increased DNMTs levels would be more susceptible to 5-aza-2′-deoxycytidine compared with those displaying low levels (Jüttermann et al., 1994). The cytotoxic effect observed is in part related to growth inhibition, G_2_ arrest, formation of DNA double-strand breaks and activation of DNA damage-related pathways (Palii et al., 2008). Likewise, since DNMT1-deficient cells showed a defective response to DNA damage and lack of DNA damage repair proteins, DNMT1 might play a significant role in DNA damage repair pathways through a DNA methylation-independent mechanism (Palii et al., 2008; Jin and Robertson, 2013).

The most well characterized nucleoside analogs, 5-azacytidine (Vidaza™), and 5-aza-2′-deoxycytidine (Dacogen™), have been widely used in pre-clinical studies and clinical trials (O'rourke et al., 2013; Graça et al., 2016). These drugs were developed in 1964 as classical cytostatic agents (Šorm et al., 1964) but in the 1980s their ability to induce cell differentiation *in vitro* and to inhibit DNA methylation was disclosed (Taylor and Jones, 1979). The clinical benefit observed in clinical trials for hematologic cancer patients (Wijermans et al., 2000; Issa et al., 2004) led to the US FDA approval of 5-azacytidine and 5-aza-2′-deoxycytidine, in 2004 and 2006, respectively, for treatment of myelodysplastic syndrome (MDS) (Kaminskas et al., 2005; Kantarjian et al., 2006). In addition to MDS, these compounds are also active against acute myeloid leukemia (AML) and other myeloid malignancies (Robak, 2011). However, both 5-azacytidine and 5-aza-2′-deoxycytidine showed limited efficacy in clinical practice, owing to their cytotoxic effect at higher doses, instability under physiological conditions and short half-life (less than 30 min) attributable to degradation by hydrolytic cleavage and deamination by cytidine deaminase (Chabner et al., 1973; Laliberte et al., 1992). Moreover, high doses of 5-azacytidine may cause neutropenia and thrombocytopenia (Braiteh et al., 2008). One of the major concerns is their lack of specificity, which might lead to unwanted activation of expression of normally silenced genes, thus contributing to tumorigenesis (Worm and Guldberg, 2002). Nevertheless, a large trial with MDS patients treated with lower doses of these agents showed not only a delay in conversion time for leukemia but also an improvement in overall survival (Fenaux et al., 2009).

To improve the stability and efficacy of azanucleosides agents, other cytidine analogs have been developed, such as zebularine, 5-Fluoro-2′-deoxycytidine, 5,6-dihydro-5-azacytidine, SGI-110, CP-4200, and Gemcitabine. Zebularine is a deoxycytidine derivative that lacks an amino group in position 4 of the pyrimidine ring (Gowher and Jeltsch, 2004). This compound stabilizes the binding of DNMTs to DNA, thereby trapping the enzyme and preventing its turnover even at other sites, resulting in decreased methylation and dissociation of the enzyme-DNA complex (Champion et al., 2010). Additionally, zebularine is stable in neutral and acidic aqueous solution, has low cytotoxicity, reduces DNA adduct formation, inhibits cytidine deaminase, and, if administered continuously, effectively maintains gene demethylation in cancer cells. This demethylating agent was also the first nucleoside analogs to reactivate an epigenetically silenced gene by oral administration (Cheng et al., 2003). In addition to the demonstrated oral bioavailability, this compound depicted a high selectivity for tumor cells (Cheng et al., 2004b).

5-Fluoro-2′-deoxycytidine (FdCyd) is a fluoropyrimidine nucleoside analog (Gowher and Jeltsch, 2004) that also forms covalent bindings with DNMTs to produce a suicide complex (Yoo and Jones, 2006; Mai and Altucci, 2009). Like zebularine, FdCyd is stable in aqueous solution, and less toxic than 5-azacytidine and 5-aza-2′-deoxycytidine (Gowher and Jeltsch, 2004). However, it is rapidly metabolized *in vivo* by cytidine deaminase (Beumer et al., 2006). To overcome this obstacle, clinical studies showed that co-administration of FdCyd with a cytidine deaminase inhibitor, such as tetrahydrouridine (THU), improved its stability (Beumer et al., 2008).

5,6-dihydro-5-azacytidine (DHAC) is a hydrolytically stable 5-azacytidine nucleoside due to the saturation of 5,6-double bond which prevents the nucleophilic attack of the position 6 by water. This compound is less cytotoxic than 5-azacytidine and 5-aza-2′-deoxycytidine overcoming their main weaknesses (Beisler et al., 1977). Similarly to 5-azacytidine, DHAC may be incorporated into RNA and inhibit its synthesis as well as DNA methylation in human cell lines (Carr et al., 1987). However, clinical trials with DHAC resulted in low response rate and significant side effects, including severe chest pain and cardiotoxicity (Carr et al., 1987; Samuels et al., 1998).

SGI-110 (guadecitabine), a dinucleotide consisting of 5-aza-2′-deoxycytidine coupled to deoxyguanosine, is highly resistant to cytidine deaminase and was shown to be effective in DNA methylation inhibition both *in vitro* and *in vivo*, although it may also act as an immune modulator (Chuang et al., 2010; Srivastava et al., 2015). Importantly, in a phase I dose-escalation study, this second-generation hypomethylating drug showed promising pharmacologic properties with good tolerance and biological activity in patients with MDS and AML (Issa et al., 2015). It was also demonstrated that this drug protects 5-aza-2′-deoxycytidine from deamination, increasing its exposure time and metabolic stability (Yoo et al., 2007; Chuang et al., 2010).

CP-4200 is an elaidic acid ester analog of 5-azacytidine that contains a fatty acid moiety to turn the cellular uptake of the drug less dependent on the nucleoside transport systems. This compound showed significantly higher efficacy compared to 5-azacytidine in an orthotopic mouse tumor model for acute lymphoid leukemia, and also a strong epigenetic modulatory effect in several human cancer cell lines (Brueckner et al., 2010).

Gemcitabine is a pyrimidine cytosine analog which acts as an anti-metabolite. This agent requires intracellular conversion into two active metabolites, gemcitabine diphosphate and gemcitabine triphosphate, which in turn may function in two ways: by binding to ribonucleotide reductase (RNR), irreversibly inhibiting it, and/or by replacing cytosine during DNA replication (Plunkett et al., 1995). This cytotoxic agent is one of the most widely used in anti-cancer therapy and is frequently employed in combination with cisplatin, forming DNA adducts that may be repaired by nucleotide excision repair (NER). The synergistic action of both drugs is thought to reside in an inhibitory effect of gemcitabine on repair of the DNA lesions induced by cisplatin (Crul et al., 2003; Moufarij et al., 2003). Interestingly, gemcitabine also displays an epigenetic effect through inhibition of NER, leading to Gadd45-mediated DNA demethylation and re-activation of epigenetically silenced genes (Schäfer et al., 2010).

A newly synthetized, orally available cytidine analog - RX-3117 (fluorocyclopentenylcytosine) - was reported to interfere with cell division, DNA synthesis, induce cell cycle arrest at G_1_ phase, and promote apoptosis. RX-3117 cellular uptake is mediated by the human equilibrative nucleoside transporter (hENT1) and requires uridine-cytidine kinase 2 (UCK2) to be activated (Sarkisjan et al., 2016). Ribonucleotide reductase (RR) reduces RX-3117 diphosphate to deoxyRX-3117 diphosphate, which may be converted to deoxyRX-3117 triphosphate and subsequently incorporated into DNA molecule, inhibiting DNMTs (Peters et al., 2013). Importantly, this drug demonstrated anti-cancer properties in xenograft models, including those resistant to gemcitabine (Choi et al., 2012; Yang et al., 2014).

#### DNMTi and “viral mimicry”

Stimulation of the immune system through upregulation of interferon-stimulated genes (ISGs) and immunoregulatory pathways were also disclosed as immunological-based mechanism of action of 5-aza-2′-deoxycytidine (Chiappinelli et al., 2015; Roulois et al., 2015). This paves the way for combination therapies associating epigenetic modulators and immunotherapeutic agents (Dear, 2016).

Recent studies have suggested that 5-aza-2′-deoxycytidine reactivates interferon-responsive genes through dsRNAs (double-stranded RNA)-containing endogenous retroviruses, which are normally silenced by promoter DNA methylation. Chiappinelli and co-workers described a mechanism that encompasses the evaluation of RNA-sensing proteins expression normally upregulated in viral infection response leading to detection of cytoplasmic dsRNA. This process triggers dsRNA-sensing pathways and subsequently the response to type I interferon (IFN), being designated “viral mimicry” (Chiappinelli et al., 2015). Type III IFNs (IL28 and IL29) are also upregulated when MAD5/MAVS/IRF7 axis is activated and induce the expression of ISGs. Furthermore, when MAD5/MAVS/IRF7 axis is genetically inactivated, cancer cell lines become insensitive to 5-aza-2′-deoxycytidine treatment. Thus, induction of a viral mimicry state in cancer cells by 5-aza-2′-deoxycytidine is dependent on the activation of MAD5/MAVS/IRF7 axis and type III IFNs (Roulois et al., 2015). Moreover, DNA demethylating agents are also implicated in T cell-mediated immune response, working in synergy with immunotherapy. Indeed, type III IFNs not only promote a robust immune response by type 1 helper CD4^+^ and CD8^+^ T cells, but are also responsible for recruitment and activation of CD4^+^ T cells after viral infection (Burkart et al., 2013; Wack et al., 2015). In an organoid culture model, treatment with 5-aza-2′-deoxycytidine induced DNA demethylation which suppressed the proliferation of intestinal tumor organoids through activation of expression of genes involved in anti-viral response, including interferon-responsive genes (Saito et al., 2016). Altogether, these findings indicate that DNMTi might modulate both innate and adaptive immune responses against cancer cells.

### Non-nucleoside analogs

The cytotoxic effects inherent to nucleoside analogs which derive from their direct incorporation into DNA (Santi et al., 1984; Yoo and Jones, 2006) entailed the discovery and development of new compounds that may directly bind to the catalytic site of DNMTs without requiring prior incorporation into the DNA molecule (Brueckner and Lyko, 2004).

Procaine (a local anesthetic) and its derivative procainamide (an anti-arrhythmic drug) are two closely related small molecules that reduce DNA methylation in cancer cells (Lin et al., 2001; Villar-Garea et al., 2003). These agents are thought to directly bind to CpG-rich sequences, perturbing the interactions between DNMTs and its target sites. Procaine was able to reduce by 40% the 5-mC DNA content and densely demethylate hypermethylated CpG islands, such as those located in the *RAR*β*2* promoter region, with concomitant re-expression of epigenetically silenced genes. Furthermore, this agent exerted growth-inhibitory effects in MCF-7 breast cancer cell line by inducing mitotic arrest (Villar-Garea et al., 2003). Procainamide preferentially inhibits DNMT1 but not DNMT3A and 3B, suggesting a more specific inhibitory effect (Lee et al., 2005). IM25 is a novel and small DNMT1 inhibitor derived from procainamide, that was shown to demethylate *GSTP1* (which encodes for a detoxifying enzyme) with lower toxicity comparatively to procainamide and 5-aza-2′-deoxycytidine Lin Y.-S. et al., 2011.

Hydralazine is a potent arterial vasodilator agent that was recognized as a demethylating agent (Segura-Pacheco et al., 2003). However, its mechanism of action is not well understood, yet. Some authors suggest that this drug inhibits DNA methylation, establishing highly stable interactions between its nitrogen atoms and the active site of DNMTs (Arce et al., 2006). Hydralazine was shown to induce demethylation and reactivation of TSG in several cancer models, without significant cytotoxic effects (Segura-Pacheco et al., 2003; Song and Zhang, 2009). Interestingly, its activity is synergized when combined with valproic acid, a short-chain fatty acid with effective HDAC inhibition properties (Arce et al., 2006; Chavez-Blanco et al., 2006; Song and Zhang, 2009).

The antibiotic nanaomycin A was reported as a selective inhibitor of DNMT3B, with the ability to reduce DNA methylation and induce re-expression of Ras-association domain family protein 1 isoform A (*RASSF1A*) TSG in cancer cell lines (Kuck et al., 2010). Most DNMTi are not specific for a certain DNMT, which may favor toxicity. To overcome this issue two novel small molecules were designed: MG98 and RG108.

MG98 is a second-generation 20-nucleotide antisense oligonucleotide designed to hybridize with the 3'-UTR of human DNMT1 mRNA leading to enzyme downregulation (Yan et al., 2003). A phase I clinical trial demonstrated that this compound not only inhibited DNMTs in a more selective manner but was also well tolerated and associated with lower cell toxicity (Plummer et al., 2009). Despite its DNMT1 inhibitory activity, this compound did not reach a significant response in clinical trials (Winquist et al., 2006; Klisovic et al., 2008; Plummer et al., 2009).

RG108 is a synthetic molecule designed to directly inhibit DNMT1 catalytic domain. This compound blocks DNMTs without causing enzyme degradation (Stresemann et al., 2006) and with low cytotoxic effects (Brueckner et al., 2005). Furthermore, RG108 has been shown to effectively reactivate several epigenetically silenced TSGs, without affecting the methylation status of centromeric repeats (Brueckner et al., 2005; Stresemann et al., 2006).

Disulfiram, a compound with strong thiol-reactive functional groups which attack the thiol group of the reactive cysteine in the active site of the aldehyde dehydrogenase enzyme (Veverka et al., 1997), is used in clinical practice for alcohol abuse treatment (Chick, 1999). This compound was described as DNMTi since it was capable of reducing global 5 mC levels, as well as demethylate and reactivate the expression of epigenetically silenced TSGs (Lin J. et al., 2011).

The quinoline-based compound SGI-1027 demonstrated inhibitory activity against DNMT1, DNMT3A and DNMT3B, possibly through interaction with the DNA substrate, resulting in demethylation and reactivation of TSGs (Datta et al., 2009; Gros et al., 2015).

Several studies suggested that non-nucleoside inhibitors are not necessarily less genotoxic and cytotoxic than nucleosides analogs. Moreover, these compounds were reported to be less effective in the inhibition of DNA methylation and reactivation of gene expression than the nucleoside analogs inhibitors (Chuang et al., 2005; Stresemann et al., 2006).

### Natural compounds

Natural products found in food might be an effective class of epigenome-targeted-drugs. These encompass epigallocatechin-3-gallate, genistein, isothiocyanates (such as phenethyl isothiocyanate), as well as curcumin. Among these, epigallocathechin-3-gallate (EGCG), a polyphenol derived from green tea, was shown to inhibit DNA methylation by binding and blocking human DNMT1 active site, leading to the reactivation of epigenetically silenced genes (Fang et al., 2003).

Genistein is one of the most common and well known isoflavones in nature and was reported as the major anti-cancer compound in soybean (Russo et al., 2016). Several molecular targets were ascribed to this compound, including estrogen receptor (*ER*) in breast cancer (Kuiper et al., 1998). Genistein was found to inhibit DNMTs, with concomitant DNA demethylation and reactivation of *BTG3, CDKN2A, MGMT*, and *RAR*β expression (Fang et al., 2005; Kikuno et al., 2008; Majid et al., 2009).

Phenethyl isothiocyanate (PEITC), found in cruciferous vegetables, is a natural compound that induces cellular growth arrest and apoptosis (Chiao et al., 2004). PEITC displayed dual action through inhibition of DNA demethylation and HDACs activity in prostate cancer (PCa) cell lines (Wang et al., 2007).

Curcumin, a polyphenolic compound (diferuloylmethane) and one of the ingredients of curry, has numerous medicinal and anti-cancer properties (Han et al., 2002). Presently, curcumin seems to be a promising chemo-preventive agent, with the ability to reverse, inhibit or prevent the development of several human malignancies, through inhibition of specific molecular signaling pathways involved in carcinogenesis (Hatcher et al., 2008; Teiten et al., 2010, 2012). Curcumin was reported to induce global hypomethylation in MV4-11 leukemia cell line through molecular docking with DNMT1, suggesting that this compound covalently blocks the catalytic thiolate of DNMT1, inhibiting DNA methylation (Liu et al., 2009).

Owing to the wide availability, tolerability/low toxicity and usual presence in the daily diet, natural compounds have been assumed to be safer than the synthetic ones. Nevertheless, most of these compounds exhibit stereogenic centers and fused ring systems which render them highly complex molecules, which simultaneously act in several targets. Their ability to interact with several protein families hinder the precise characterization of its anti-neoplastic effects, making it difficult to discriminate whether these result from direct modulation of one or more epigenetic targets. This unspecific mode of action might culminate in activation of unwanted pathways that may favor tumor growth and progression (Miceli et al., 2014; Aggarwal et al., 2015). In an attempt to overcome this, chemical probes have been progressively highlighted in recent years as valuable small-molecules to investigate complex biological mechanisms and processes related with disease onset, including cancer. Moreover, among the several promising features of high-quality chemical probes, selectivity to relevant molecular targets in a cancer model might assist in discovery of novel drugs from chemical molecule libraries (Blagg and Workman, 2017).

## DNMTi in urological cancers: pre-clinical and clinical trials

**Figure 3:**
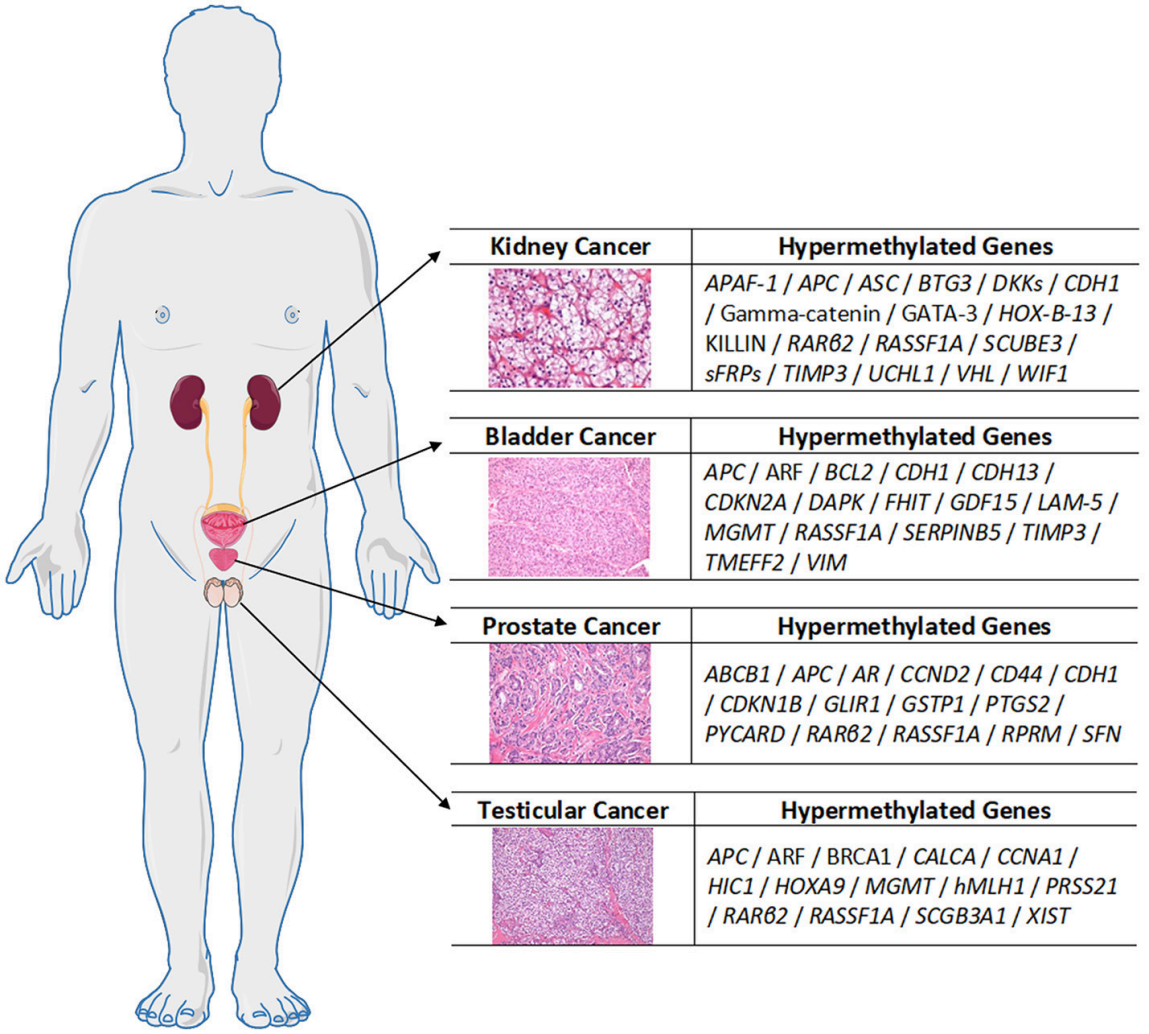
An overview of the most relevant hypermethylated genes in each urological tumor, compiled from the referenced cited in the main text.

Urological cancers are known to harbor epigenetic alterations, with hypermethylation of TSGs as one of the most studied alterations, along with histone post-translation modifications. The panel of the most relevant hypermethylated genes in each urological tumor type is depicted in Figure 3. Over the last decades, several reports highlighted the clinical usefulness of DNMTi for therapy of urological cancers. Except for PCa, however, the role of these agents in urological cancer treatment is still rather unexplored.

Pre-clinical and clinical studies performed for each urological cancer type are reviewed in the next sections, and Table 1 provides a summary of all completed, terminated and ongoing clinical trials.

**Table 1:** Clinical trials of DNMTs inhibitors for urological tumors.

**Cancer**	**Drug**	**Phase (ID)**	**Status**	**Schedule**	**Outcome**	**Ref**.
**Bladder**	5-Aza-2'-deoxycytidine	I (NCT00030615)	Completed	Advanced BlCa patients (*n* = 24) received daily escalating doses of 5-aza-2'-deoxycytidine for 4 weeks. Courses repeated every 6 weeks in the absence of disease progression or unacceptable toxicity.	Data not available.	ClinicalTrials.gov, 2002
	CC-486	I (NCT02223052)	Recruiting (estimated completion date: 5 September 2018)	Patients with hematologic or solid malignancies including genitourinary tumors (*n* = 90) have been enrolled to evaluate bioequivalence and food effect bioavailability of CC-486 (oral azacytidine).	Data not available.	ClinicalTrials.gov, 2014
	CC-486 with Carboplatin or Paclitaxel protein bound particles (ABI-007)	I (NCT01478685)	Completed	CC-486 was administered orally at doses between 100-300 mg daily for either 14 or 21 days. Carboplatin was given by intravenous (i.v.) infusion once every 21 days. ABI-007 was administered by i.v. infusion on two of every three weeks at a dosage of 100 mg/m^2^.	Data not available.	ClinicalTrials.gov, 2011
	5-Azacytidine and Sodium phenylbutyrate	I	Completed	5-azacytidine subcutaneously (s.c.) and sodium phenylbutyrate (continuous i.v. infusion) were administered in refractory BlCa (*n* = 2), RCC (*n* = 3) and PCa (*n* = 5) patients regarding three different dose regimens during 48 cycles in 27 advanced solid tumors patients.	The results were disappointing for genitourinary malignancies, since none of the patients responded to the treatment. From all enrolled patients, only one exhibited stable disease for 5 months whereas 26 patients showed progressive disease. Grade 3 and 4 neutropenia was observed.	Lin et al., 2009
	FdCyd and THU	II (NCT00978250)	Recruiting (estimated completion date: 2 June 2020)	Both drugs will be administrated over 28-day cycles through a vein for about 3 h each day on days 1, 5, and 8, 12 of each cycle. Patients may continue to receive FdCyd and THU if their cancers does not grow, if they do not have too many side effects, and if they are willing to do so.	Data not available.	ClinicalTrials.gov, 2009b
	RX-3117	I/II (NCT02030067)	Recruiting (estimated completion date: December 2017)	Phase I study intend to determine the maximum tolerated dose of RX-3117. The enrolled subjects (*n* = 72) will be treated in a dose expansion followed by a 2-stage Phase 2 study for over 8 cycles of therapy. Each cycle will last 4 weeks. RX-3117 will be administered 3 times each week for 3 weeks follow by one-week rest. All subjects will be followed for at least 30 days after the last dose.	Data not available.	ClinicalTrials.gov, 2013
	MG98	I	Completed	Patients with advanced solid tumors (*n* = 33), including bladder and prostate cancer were treated with escalating doses of MG98 in an i.v. infusion over 7 days every 14 days.	Patients with genitourinary tumors did not respond to the treatment. Mild toxicities including fatigue, headache, and myalgia grade ≤2.	Plummer et al., 2009
	75 approved agents	II (NCT02788201)	Recruiting (estimated completion date: 29 June 2019)	Evaluate whether the Co-expression Extrapolation (COXEN) model can chose the best therapeutic option for advanced urothelial carcinoma within 3 weeks. Patients whose BlCa has spread after at least one chemotherapy line (**n** = 20) are estimated to be enrolled. The subjects will take the drugs assigned by the COXEN model and will have regular visits for blood and urine tests as well as tumor scans.	Data not available.	ClinicalTrials.gov, 2016a
**Kidney**	5-Aza-2'-deoxycytidine	I	Completed	Refractory RCC patients (*n* = 3) received intravenously 5-aza-2'-deoxycytidine from 2.5 to 10 mg/m^2^ on days 1-5, and from 8 to 12, or 15 to 20 mg/m^2^ on days 1–5. Each cycle lasted four weeks.	Relative reduction of tumor size; Increased tumor apoptosis; Reduction of DNA methylation in both tumor and PBMC but without correlation between them; Increased expression of CTR1.	Stewart et al., 2009
	5-Aza-2'-deoxycytidine and Interleukin-2	I	Completed	Renal cancer patients (*n* = 5) received subcutaneous 5-aza-2'-deoxycytidine daily x 5 days on weeks 1 and 2 of a 12-week cycle. High-dose IL-2, consisting of two cycles of IL-2 600,000 IU/kg intravenously every 8 hours' x 14 doses separated by a 2-week break, was administered starting on week 3. Decitabine was escalated from 0.1 to 0.25 mg/kg.	Global DNA demethylation. Up-regulation of immunomodulatory genes. Three of five evaluable patients presented stable disease. Grade 4 neutropenia was observed.	Gollob et al., 2006
	5-Aza-2'-deoxycytidine and Interferon alfa-2β	II (NCT00561912)	Terminated	Patients with advanced RCC (*n* = 2) received 5-aza-2'-deoxycytidine 15 mg/m^2^ intravenously daily over 1h for 5 days plus Interferon Alfa-2b 0.5 million units subcutaneously twice daily continuously, on day 1 cycle 3. Each cycle was 28 days long.	Terminated due to low accrual.	ClinicalTrials. ClinicalTrials.gov, 2007
	5-Aza-2'-deoxycytidine and Anti-PD-1	I/II (NCT02961101)	Recruiting (estimated completion date: May 2019)	Patients with relapsed or refractory malignancies, including RCC, will be enrolled to evaluate the feasibility, safety and efficacy of an anti-programmed cell death protein 1 (PD-1) antibody combined with low-dose 5-aza-2'-deoxycytidine every 3 weeks.	Data not available.	ClinicalTrials.gov, 2016b
	5-Azacytidine and Valproic Acid	I	Terminated	5-Azacytidine was administered subcutaneously daily for 10 days in patients with advanced cancers (*n* = 55), two of them with RCC. Cycles were 28 days long and 5-azacytidine was administered at 75 mg/m^2^.	One of the two treated RCC patients achieved a 6 months' stable disease. Data not available for decrease in methylation but it was observed histone acetylation. Grade 3 and 4 toxicities were observed.	Braiteh et al., 2008
	5-Azacytidine and Interferon-α-2β	I (NCT00217542)	Completed	Patients (*n* = 42) received azacytidine subcutaneously once daily on days 1–4 and 15–17 and recombinant interferon alfa-2b subcutaneously on specific days during course 1. Beginning in course 2 and for all subsequent courses, patients received azacytidine subcutaneously once daily on days 1–3 and 15–17 and interferon alfa-2b subcutaneously on specific days. Treatment repeated every 28 days for up to 12 total courses in the absence of disease progression or unacceptable toxicity.	Data not available.	ClinicalTrials.gov, 2005
	5-Azacytidine and Bevacizumab	I/II (NCT00934440)	Terminated	All patients with advanced RCC (*n* = 23) in phase I and II received bevacizumab at the standard dose of 10 mg/kg every 2 weeks and the doses were administered at specific times. 5-azacytidine was administered in different dose levels for each study phase (I/II).	Data not available.	ClinicalTrials.gov, 2009a
	MG98	I/II (NCT00003890)	Completed	Untreated patients (*n* = 17) with measurable metastatic renal carcinoma received MG98 360 mg/m^2^ intravenous over 2 h twice weekly for 3 weeks. Courses were repeated every 4 weeks.	No conclusive pattern of decreased DNMT1 activity was detected after MG98 treatment. Toxicities was experienced including rigors, fatigue, fever, and nausea.	Winquist et al., 2006
	MG98 and Interferon-α-2β	Study phase not provided	Completed	Patients with advanced RCC (*n* = 19) were divided in 2 groups: 10 received a continuous regimen and 9 received an intermittent regimen twice weekly. In the first group, patients received MG98 in two 7-day continuous infusions every treatment week followed by a week of rest in each cycle. In the intermittent group, patients were treated with a 2h intravenous infusion of MG98 twice per week for three weeks with the last cycle week of rest. Additionally, both groups received interferon-α-2β subcutaneously three days per week with an initial dose of 12 MIU/day or 9 MIU/day.	Interferon-α-2β 9 MIU plus MG98 125 mg/m^2^ for a continuous schedule and interferon-α-2β 9 MIU plus MG98 200 mg/m^2^ for an intermittent schedule were considered the maximum tolerable doses. The first showed 2 out of 7 dose-limiting grade 3 toxicities, including fever and thrombocytopenia. One partial response and eight stable disease were achieved.	Amato et al., 2012
**Prostate**	5-Azacytidine, docetaxel and prednisone	I/II (NCT00503984)	Terminated	mCRPC cancer patients who progressed during or within 6 months of docetaxel chemotherapy, were eligible (*n* = 22). In phase I, patients received the highest dose of azacytidine 150 mg/m^2^ daily for 5 days + Docetaxel 75 mg/m^2^ on day 6. In phase II, it was used the combination of azacytidine 75 mg/m^2^ daily for 5 days followed by docetaxel 75 mg/m^2^ on day 6 along with growth factor support and fixed prednisone 5 mg since day 1 to 21.	In phase I, grade 4 neutropenia was frequent. In phase II, 10 of 19 evaluable patients showed PSA response and 3 of 10 achieved an objective response. Significant demethylation of GADD45A was observed.	Singal et al., 2015
	5-Azacytidine and Combined Androgen Blockade (CAB)	II (NCT00384839)	Completed	Chemonaïve patients with CRPC on CAB and PSA-doubling time (DT) < 3 months were eligible (*n* = 36). CAB was continued and 5-azacytidine 75 mg/m2 was administered for 5 consecutive days of each 28-day cycle up to 12 cycles or until clinical progression or intolerable toxicities.	19 patients attained a PSA-DT ≥3 months. Overall median PSA-DT was significantly prolonged with 2.8 months. The obtained median clinical progression-free survival was 12.4 weeks. Grade 3 toxicities of fatigue and neutropenia were observed.	Sonpavde et al., 2011
	5-Azacytidine and Valproic Acid	I	Completed	5-Azacytidine was administered subcutaneously daily for 10 days in patients with advanced cancers (*n* = 55), in which two of them with PCa. Cycles were 28 days long and 5-azacytidine was administered at 75 mg/m^2^.	One of the two treated PCa patients achieved a 6 months' stable disease.	Braiteh et al., 2008
	5-Azacytidine and Sodium Phenylbutyrate	II (NCT00006019)	Completed	Patients (*n* = 20) received 5-azacytidine on days 1–7 and phenylbutyrate i.v. over 1–2 h on days 8–12. Additional courses in these patients were repeated every 21–28 days in the absence of disease progression or unacceptable toxicity.	Data not available.	ClinicalTrials.gov, 2000
	5-Aza-2'-deoxycytidine	II	Completed	Patients with metastatic recurrent PCa after total androgen blockade and flutamide withdrawal (*n* = 14) received an infusion of 3 doses of 5-aza-2'-deoxycytidine 75 mg/m^2^. Cycles of therapy were repeated every 5–8 weeks to allow for resolution of toxicity.	Two of 12 patients evaluable for response had stable disease with a time to progression of > 10 weeks. 5-Aza-2'-deoxycytidine was well tolerated with modest clinical activity against CRPC.	Thibault et al., 1998
	SGI-110 and Pembrolizumab	I (NCT02998567)	Not yet recruiting	Patients with refractory solid tumors (*n* = 35), including CRPC, will receive s.c. SGI-110 daily on days 1–4 of each 21-day cycle. Pembrolizumab will be administered i.v. once per 21-day cycle on specific days.	Data not available.	ClinicalTrials.gov, 2016c
	Curcumin and Radiotherapy	Study phase not provided (NCT01917890)	Completed	PCa patients (*n* = 40) treated with external beam radiotherapy were separated in two groups 20 of them received 3 g/day curcumin orally and 20 received placebo.	Patients treated with curcumin presented reduced urinary symptoms, showing possible radioprotective effects.	Hejazi et al., 2013
	Disulfiram	I (NCT01118741)	Completed	Eligible patients (*n* = 19) were ≥18 years old, previously treated with local therapy and subsequently developed biochemically recurrent disease. Cohort 1 (*n* = 9) and 2 (*n* = 10) received disulfiram treatment 250 mg and 500 mg daily, respectively. The primary endpoint was the proportion of subjects with a demethylation response. Secondary endpoints included rate of PSA progression at 6 months, changes in PSA doubling time and safety/tolerability.	Only five of the evaluable subjects were on trial for ≥ 6 months from cohort 1 and obtained a PSA progression by 6 months. Three of the responders displayed pretreatment instability in their 5-mC content. Six patients experienced grade 3 toxicities.	Schweizer et al., 2013
**Testicular**	5-Azacytidine	II	Completed	Patients with solid tumors (*n* = 214) were enrolled and four of them were testicular cancer patients. 5-azacytidine doses varied from 225 mg/m^2^ to 150 mg/m^2^.	Two of the four testicular cancer patients presented partial responses.	Quagliana et al., 1976
		II	Completed	Patients (*n* = 17) received 5-azacytidine at a dosage of 150 mg/m^2^/day at days 1 to 5 by continuous infusion every three weeks.	It was not observed any 5-azacytidine activity. Grade 3 and 4 toxicities were reported.	Roth et al., 1993
	SGI-110 and cisplatin	I (NCT02429466)	Recruiting (estimated completion date: 31 December 2018)	TGCT patients (*n* = 15) who relapsed after several chemotherapy lines will be enrolled. SGI-110 will be given subcutaneously, daily, 30 mg/m^2^ on days 1–5 followed by cisplatin 100 mg/m^2^ on day 8, every 4 weeks. Treatment will be continued for a maximum of 6 cycles or until disease progression or unacceptable toxicity.	Data not available.	ClinicalTrials.gov, 2015
	Hydralazine and Magnesium Valproate	II (NCT00404508)	Completed	Patients with refractory solid tumors (*n* = 15) received hydralazine at 182 mg for rapid, or 83 mg for slow, acetylators, and magnesium valproate at 40 mg/kg, beginning a week before chemotherapy.	A decrease in chemotherapy resistance was observed. A clinical benefit was reported, namely stable clinical response a 5.6 months progression-free survival and an overall survival of 5.7 months	Candelaria et al., 2007

### Bladder cancer

Bladder cancer (BlCa) is the 9th most common malignant tumor worldwide and its incidence is 3 times higher among men than women, representing the 4th and the 11th most common cancer, respectively (Antoni et al., 2016). However, for unknown reasons, women have higher mortality rates than men (Burger et al., 2013).

Urothelial cell carcinoma (UCC) is the most common BlCa histological subtype representing about 90% of all cases, whilst the remaining 10% are non-urothelial subtypes, including squamous cell carcinoma and adenocarcinoma. UCC can be further classified into papillary (low-grade papillary and high-grade papillary carcinomas) and invasive (Solomon and Hansel, 2016) subtypes. Although more than 80% of UCC are superficial lesions with a favorable prognosis, these tend to recur frequently. This relapsing nature of BlCa makes it one of the most expensive human malignancies to treat (Avritscher et al., 2006). On the other hand, invasive BlCa is often correlated with fatal outcome (Kaufman et al., 2009). Low-grade UCC is primarily managed by bladder-sparing techniques as endoscopic resection and intravesical chemotherapy. Whereas in muscle-invasive BlCa, radical cystectomy with neoadjuvant chemotherapy is first line treatment, patients with advanced disease are treated with systemic cisplatin-based chemotherapy regimens (Kamat et al., 2016). However, these therapies are mostly not curative, and this aggressive phenotype can develop resistance to chemotherapy resulting in treatment failure. Furthermore, 5-year survival rate of patients with locally advanced or metastatic BlCa is lower than 25% (Drayton and Catto, 2012), emphasizing the need for new therapeutic approaches at the more advanced disease stages.

Concerning epigenetic alterations, high-grade UCCs disclose higher hypermethylation levels and upregulation of several microRNAs (miRNAs), compared with low-grade UCCs (Yates et al., 2007; Catto et al., 2009). The main difference between low- and high-grade UCC seems to be the amount of aberrant hypermethylation instead of the specific targets. Epigenomic profiling has revealed that in low-grade UCC, 10% of the loci displayed aberrant DNA methylation patterns, in contrast with high grade noninvasive and invasive UCC which display over 20% and 30% methylated loci, respectively (Catto et al., 2005; Dhawan et al., 2006; Wolff et al., 2010). Biallelic expression of the imprinted gene *IGF2* and imprinted maternally expressed transcript *H19* were found in about 20% of BlCa (Byun et al., 2007). Hypomethylation of *LINE-1* gene was correlated with increased risk of BlCa and could be a potential biomarker for BlCa diagnosis and treatment (Wilhelm et al., 2010). Several genes have been reported to be hypermethylated in BlCa, including *APC, ARF, CDKN2A, DAPK, LAM-5* and *RASSF1A*, and have also been proposed as detection markers for BlCa (Chan et al., 2002; Dulaimi et al., 2004b; Friedrich et al., 2004). Additionally, high *APC, CDH1, CDH13, FHIT, RASSF1A* promoter methylation levels correlated with poor prognosis, adverse clinicopathological features, disease progression, and overall survival (Maruyama et al., 2001; Yates et al., 2007). Corroborating these data, *APC* and *RASSF1A* along with *MGMT* hypermethylation were associated with high-grade and invasive tumors in patients with BlCa (Bilgrami et al., 2014). *SERPINB5* (gene that encodes for maspin) expression is higher in normal urothelium, preserved in superficial BlCa, but it is significantly diminished in invasive carcinomas. This low maspin expression is correlated to gene hypermethylation and with increased tumor cell growth *in vivo* (Beecken et al., 2006; Zhang et al., 2013). Moreover, a genomewide approach, led to identification of three genes panel, *GDF15, TMEFF2* and *VIM*, that accurately detect this neoplasm (Costa et al., 2010). Furtermore, *BCL2* methylation levels were significantly associated not only with disease stage, but also with tumor grade (Friedrich et al., 2004).

#### Pre-clinical studies

Regarding BlCa, most pre-clinical assays evaluated the efficacy of nucleoside inhibitors as potential anti-cancer agents, both in monotherapy or in combination with other agents. BlCa cells exposure to 5-azacytidine induced DNMT3A/3B expression downregulation, *hepaCAM* re-expression, being able to reduce bladder tumor growth in nude mice. In addition, 5-azacytidine inhibited BlCa cells proliferation, and G_0_/G_1_ phase cell cycle arrest (Wang et al., 2016). The treatment of 19 dogs with spontaneous urothelial BlCa, disclosed moderate myelosuppression of the testing animals. Globally, partial remission was achieved in 22%, 50% showed stable disease, whereas in 22% disease progressed (Hahn et al., 2012).

Recently, non-cytotoxic nanomolar 5-aza-2′-deoxycytidine concentrations (0.1–1 μM) were used in BlCa cell lines (HT1376, T24, B01, and B02). NOTCH1 was activated at both transcript and protein levels and CK5-dependent differentiation was induced. Notably, ICN1 (the active intracellular domain of NOTCH1) expression was shown increase after drug exposure, resulting in significant inhibition of cell proliferation (greater than 50%) without affecting cell viability. Significant changes in cell size leading to senescence-like morphological alterations were also observed. These changes were associated with substantial IL-6 release. Overall, in muscle-invasive BlCa cell lines, 5-aza-2′-deoxycytidine promoted cellular differentiation through NOTCH1 signaling and increase of IL-6 release (Ramakrishnan et al., 2017). Exposure of T24 cells to different concentrations of 5-aza-2′-deoxycytidine resulted in increased levels of maspin mRNA and protein in a dose dependent manner. In addition, proliferation, migration and invasion of T24 cells were significantly inhibited, whereas the apoptosis was greatly increased. All these effects were associated with the activation of caspase-3, decreased ratio of Bcl-2/Bax, and reduced expression of cyclin D1, VEGF-C, MMP-2, and MMP-9 (Zhang et al., 2013). B-cell translocation gene 2 (*BTG2*) is downregulated in human BlCa and this might be associated with DNMT1 bind to *BTG2* locus, suppressing gene expression through downregulation of its transcription factor, Sp1. 5-aza-2′-deoxycytidine induced *BTG2* demethylation and re-expression by inhibiting DNMT1 expression. Thus, increased *BTG2* expression significantly reduced the tumorigenic and invasive capabilities *in vitro* of the highly malignant EJ cells, together with induction of G_2_/M arrest (Devanand et al., 2014). Additionally, 5-aza-2′-deoxycytidine suppressed cellular growth in several BlCa cell lines. In fact, this drug induced demethylation and upregulation of *p16* gene which was associated with G_1_ cell cycle arrest. Importantly, exposure of T24 cells to 5-aza-2′-deoxycytidine prior to injection into nu/nu mice decreased the rate of tumor growth and lead to reactivation of *CDKN2A* (Bender et al., 1998).

A comparative study with 5-aza-2′-deoxycytidine and zebularine reported that these two agents equally retarded the cell growth of BlCa cell lines (T24 and RT4). Contrarily to T24 cells, whose exposure to zebularine resulted in the same proportion of cell doubling-time (DT) extension as 5-aza-2′-deoxycytidine (79.8 vs. 79.6%, respectively), in RT4 cells the DT was higher when treated with 5-aza-2′-deoxycytidine (66.4 vs. 17.5%). Moreover, these two compounds diminished the methylation index and concomitantly re-expressed *APAF-1* (Christoph et al., 2006).

A pre-clinical *in vivo* study with single-agent SGI 110 and 5-aza-2′-deoycytidine in murine xenograft models derived from BlCa cell lines was conducted. Both intraperitoneal and subcutaneous administration of the drugs effectively reduced *CDKN2A* promoter methylation levels, inducing its expression, and inhibited murine tumors growth. However, the treatment was not sufficient to reduce tumor size and subcutaneous delivery revealed a more favorable toxicity profile compared with intraperitoneal route (Chuang et al., 2010).

Considering all these reports, hypomethylating compounds seem to be more effective in BlCa cell lines with hypermethylated *CDKN2A*/p16 such as T24 and its clone EJ.

#### Combined therapy

5-Azacytidine combined with the histone deacetylase inhibitor Trichostatin A (TSA) decreased cell proliferation by attenuating the expression of DNMT1 in canine invasive urothelial carcinoma cells. Additionally, this combination caused a noticeable increase in p16 protein expression through demethylation of its gene promoter region (Dhawan et al., 2013). A recent pre-clinical study showed a unidirectional cross-resistance of cisplatin-resistant UMUC3 cells to docetaxel. However, pre-treatment of BlCa cell lines (UMUC3, T24, and TCSSUP) with 5-azacytidine resulted in enhanced sensitivity to chemotherapeutic drugs (cisplatin and docetaxel) through demethylation and up-regulation of epigenetically silenced genes involved in apoptotic pathways (Ramachandran et al., 2011).

The combination of 5-aza-2′-deoxycytidine with cisplatin inhibited proliferation through inducing G_2_/M cell cycle arrest and apoptosis of UCC cell lines. Moreover, 5-aza-2′-deoxycytidine enhanced not only the cisplatin-induced upregulation of caspase activity but also its anti-proliferative effect by increasing the population of cells at sub-G_1_ and G_2_/M phases (Shang et al., 2008). Recently, a study involving several BlCa cell lines reported that BlCa cells resistant to cisplatin chemotherapy with high *HOXA9* promoter methylation levels were sensitized to cisplatin after treatment with 5-aza-2′-deoxycytidine alone or in combination with the HDAC inhibitor vorinostat (Xylinas et al., 2016). Interestingly, the combination of four drugs (gemcitabine, cisplatin, 5-aza-2′-deoxycytidine and TSA), inhibited the canonical Wnt/β-catenin pathway and decreased cell proliferation through repression of DNA methylation. Furthermore, the anti-apoptotic gene *BCL2L1* was significantly downregulated (Varol et al., 2014).

Exposure of BlCa cell lines to zebularine resulted in effective *CDKN2A* demethylation along with depletion of DNMT1. Sequential exposure of these cells to an initial dose of 5-aza-2′-deoxycytidine (1 μM) followed by zebularine (50 μM) prevented *CDKN2A* re-methylation and re-silencing over extended time periods (Cheng et al., 2004a).

#### Clinical trials

A phase I trial which enrolled 33 patients with solid malignancies, including bladder and prostate cancers, aimed to evaluate MG98 safety and efficacy when administered in an infusion over 7 days repeated every 14 days. Whereas dose-limiting toxicities corresponded to grade 3 thrombocytopenia and transaminitis, treatment-related toxicities were mostly mild and included fatigue, headache and myalgia of grade ≤2. Unfortunately, the treatment response was only observed in patients that did not present genitourinary tumors (Plummer et al., 2009).

Patients with refractory solid tumors have been co-treated with 5-azacytidine and sodium phenylbutyrate in a phase I study. The results were disappointing for patients with genitourinary malignancies (bladder, kidney, and prostate cancers) as they did not reveal a clinical response (Lin et al., 2009). Other clinical trials testing demethylating drugs in BlCa are ongoing and still in the recruiting phase (Table 1).

### Kidney cancer

Renal cell carcinoma (RCC) stands amongst the 10 most common malignancies in the developed countries, with increasing incidence rates. The disease is more frequent in men, with a male: female ratio of 1.5:1.0 (Torre et al., 2015). RCC is the most common type of kidney cancer (~85%) and disclose an heterogeneous histology, genetics and clinical behavior (Lopez-Beltran et al., 2009). Benign tumors comprise papillary adenoma, oncocytoma, metanephric adenoma, and adenofibroma (Lopez-Beltran et al., 2009). Malignant tumors are classified into three most common subtypes: clear cell renal cell carcinoma (ccRCC) (65–70%), the most aggressive phenotype, papillary renal cell carcinoma (pRCC) (15–20%), and chromophobe renal cell carcinoma (chRCC) (5–10%), the less aggressive subtype (Moch et al., 2016). At early disease stages, RCC is clinically silent (Lam et al., 2005). However, owing to the widespread use and improved sensitivity of imaging techniques, the incidental detection of small and low-stage RCCs has increased. Nevertheless, locally advanced disease and distant metastasis are still diagnosed in a sizeable proportion of patients (Siegel et al., 2015; Capitanio and Montorsi, 2016). Surgery is the standard treatment for RCC (Capitanio and Montorsi, 2016). Advanced RCC are highly resistant to conventional chemotherapy partly due to P-glycoprotein (*P-gp*) overexpression, which occurs in 76% of the tumors (Amato, 2000; Walsh et al., 2009). Therefore, targeted therapy with tyrosine kinase inhibitors (TKIs) is currently first-line therapy for advanced RCC (Capitanio and Montorsi, 2016). Unfortunately, it is not curative and eventually all patients will become resistant to TKIs, with a dismal prognosis as metastatic RCC entails a 5-year survival rate of only 5–10% (Motzer and Russo, 2000; Duran et al., 2016).

A DNA methylation and transcriptome profiling of several RCC histological subtypes revealed a 3-fold increase in hypermethylation for ccRCC, pRCC, translocation RCC, as well as mucinous and spindle cell carcinomas compared to chRCC and oncocytoma (Malouf et al., 2016). DNMT1, DNMT3A, and DNMT3B proteins were overexpressed in the most frequent sporadic human RCC subtypes and correlated with clinicopathological stage, as well as shorter overall and disease-free survival (Li et al., 2014). These data are consistent with a recent study that reported a considerable reduction of global genome 5 hmC levels in ccRCC comparatively with non-cancerous renal tissue (Munari et al., 2016). In about 11–30% of sporadic RCC, *VHL* function is lost due to hypermethylation of a CpG island in its promoter region (Herman et al., 1994; Clifford et al., 1998; Sato et al., 2013). A common feature of ccRCC subtype is *VHL* inactivation through gene mutation, deletion, and/or promoter methylation. A study reported that within 226 ccRCC cases, 97.8% had altered *VHL* allele being 11% of those due to promoter hypermethylation. ccRCC was also linked to frequent mutations in genes related with chromatin modifications (*BAP1, KDM5C, KDM6A, PBRM1*, and *SETD2*; Sato et al., 2013). *RASSF1A* silencing due to promoter methylation was proposed as a marker not only for early detection, but also for surveillance and disease monitoring (Peters et al., 2007). This gene promoter was found methylated in 59% of ccRCC and 75% of pRCC (Morris and Maher, 2010). The *ubiquitin carboxyl-terminal esterase 1* (*UCHL1*) gene, a TSG involved in regulation of cellular differentiation, is silenced by promoter hypermethylation in RCC patients, correlating with poor prognosis (Kagara et al., 2008). Likewise, the pro-apoptotic gene *ASC/TMS1* was found downregulated by promoter hypermethylation in six RCC cell lines and in 41% of RCC tumors compared to controls. Importantly, hypermethylation of this gene was correlated with higher nuclear grade, having potential as a diagnostic and therapeutic biomarker (Liu et al., 2015). The high methylation frequency of Wnt antagonists family (*sFRPs, DKKs*, and *WIF1*) in serum DNA from RCC patients was associated with higher tumor grade, suggesting that these genes might be putative progression markers (Urakami et al., 2006). A genome-wide analysis identified nine candidate genes with frequent promoter region methylation in primary RCC tumor samples. Among those, the methylation status of *SCUBE3* was associated with a significantly augmented risk of relapse or cancer death (Morris et al., 2011). Notably, numerous TSGs were also reported to be hypermethylated in RCC samples compared to normal tissue (*APAF-1, APC, BTG3, CDH1, Gamma-catenin, GATA-3, HOX-B-13, KILLIN, RAR*β*2, RASSF1A, TIMP3, VHL*, and others), associating with increased tumor cell proliferation, invasion, and metastization (Morrissey et al., 2001; Nojima et al., 2001; Dulaimi et al., 2004a; Hoque et al., 2004; Shinojima et al., 2006; Shenoy et al., 2015).

#### Pre-clinical studies

Fifteen RCC cell lines exposed to 5-azacytidine disclosed significant decrease in cell proliferation, correlating with changes in *VHL* promoter methylation. However, a response was also observed in cell lines without *VHL* promoter methylation, suggesting a role for other hypermethylated TSGs, re-activated after DNMTi exposure. Among those, *GCM2, NEFM*, and *RGS7* displayed the most significant association with poor prognosis (Ricketts et al., 2013).

5-aza-2′-deoxycytidine suppressed the canonical Wnt/β-catenin pathway and induced apoptosis of Caki-2 cell line through demethylation and re-expression of *sFRP2* and downregulation of p-GSK3β protein (Konac et al., 2013). This DNMTi was also able to demethylate and re-express *ABCG2* and *ASC/TMS1* genes in RCC cell lines (To et al., 2006; Liu et al., 2015). Recently, 5-aza-2′-deoxycytidine was shown to decrease proliferative activity of several RCC cell lines (ACHN, Caki-1, Caki-2, and A-498) mostly through induction of cell cycle arrest at G_2_/M in a dose-dependent manner. This effect might be related with the suppression of p38-NF-kB pathway phosphorylation accomplished by this demethylating agent (Shang et al., 2015). Exposure of *RAR*β*2*-negative primary cell lines (RCC1.18) to 1 μmol/L 5-aza-2′-deoxycytidine for 96 h re-activated *RAR*β*2* expression (Wang et al., 2005). 5-Aza-2′-deoxycytidine also re-expressed *VHL* both in RCC cell lines and in xenograft murine tumors, significantly reducing tumor size of ccRCC xenograft in mice. Moreover, only tumors with *VHL* promoter methylation disclosed a treatment-related response (Alleman et al., 2004). The exposure of RCC cell lines to 5-aza-2′-deoxycytidine induced reactivation of the pro-apoptotic *RASSF1A* gene silenced by promoter hypermethylation (Dreijerink et al., 2001). This compound was also able to induce mRNA and protein expression of *UCHL1* gene in several RCC cell lines (Seliger et al., 2009).

RCC cell line A498 exposed to low-cytotoxic doses of zebularine induced a limited inhibition of cell proliferation, not superior to 40%. On the other hand, 308 genes transcripts were found upregulated at least 1.5-fold. Namely, metallothionein family genes (strong protectors against oxidative stress) were re-expressed after treatment with low-dose zebularine (Alkamal et al., 2015).

SGI-110 induced expression of cancer testis antigen (CTA)-related genes (*MAGE-A1, MAGE-A2, MAGE-A3, MAGE-A4, MAGE-A10, GAGE 1-2, GAGE 1-6, NY-ESO-1*, and *SSX1-5*) at both transcript and protein levels. Additionally, this agent upregulated human leukocyte antigen (HLA) class I antigens and intracellular adhesion molecule (ICAM-1). Altogether, these results I indicate that SGI-110 may potentiate immunogenicity of RCC and other cancer cell lines, contributing to improved recognition of cancer cells by gp-100 specific cytotoxic T lymphocytes (Coral et al., 2013).

A green tea extract composed by flavan-3-ols and EGCG, strongly inhibited the growth of A-498 and 769-P cell lines in a concentration-dependent manner, disclosing promising anti-cancer properties for RCC (Carvalho M. et al., 2010). Recently, EGCG also demonstrated therapeutic effect in RCC by inhibiting cell proliferation, inducing apoptosis and suppressing both cells migration and invasion by downregulation of both matrix metalloproteinase-2 (MMP-2) and matrix metalloproteinase-9 (MMP-9) (Chen et al., 2016).

Interestingly, oral administration of RX-3117 displayed anti-tumor effect in Caki-1 xenografts, decreasing cell proliferation in a dose-dependent manner. Moreover, RX-3117 showed higher efficient than gemcitabine's (Yang et al., 2014).

#### Combined therapy

A recent report, that evaluated the methylation profile of drug target genes in RCC, demonstrated that 1 μM of 5-aza-2′-deoxycytidine decreased 5-mC content in genomic DNA of Caki-2 cells leading to organic cation transporter 2 (encoded by *SLC22A2* gene) re-expression. Moreover, exposure to this compound resulted in demethylation and upregulation of 33 out of the 55 tested SLC drug transporters. As these transporters are associated with sensitivity to platinum chemotherapy, the combination of 5-Aza-2′-deoxycytidine with cisplatin was investigated. Remarkably, this drug combination was more effective in apoptosis induction than either drug alone (Winter et al., 2016). Treatment of murine renal cell carcinoma (Renca) cells with 5-aza-2′-deoxycytidine resulted in re-expression of TβR-II at both mRNA and protein levels. This allowed for restoration of Renca cells' sensitivity to TGF-β through an increase in phosphorylation of Smad2, which is a consequence of TGF-β receptors activation (Zhang et al., 2005). Recently, significantly induced global genomic demethylation of RCC cells, with restoration of *APAF-1* expression at both mRNA and protein levels, due to 5-aza-2′-deoxycytidine, was demonstrated. Moreover, 5-aza-2′-deoxycytidine promoted the apoptotic effect of cisplatin in ACHN cells (Zhu et al., 2014). Interestingly, exposure of RCC cell lines (A498 and CCa-5) to 5-aza-2′-deoxycytidine and zebularine effectively inhibited tumor cell growth and re-expressed *APAF-1* and *DAPK-1* mRNA. Remarkably, zebularine was more effective in achieving DT prolongation than 5-aza-2′-deoxycytidine in RCC cells (132 vs. 106% for CCa-5; 54.6 vs. 21.9% for A498) (Christoph et al., 2006). The natural compound Genistein used in combination with 5-aza-2′-deoxycytidine significantly decreased *BTG3* (TSG) promoter methylation reactivating gene expression. Furthermore, this combination decreased DNMTs and methyl-CpG-binding domain 2 (MBD2) binding and increased histone acetylation (Majid et al., 2009). Exposure of human pRCC ACHN cell line to 5-aza-2′-deoxycytidine synergistically increased the anti-proliferative effects of IFN-α e IFN-β. In addition, this compound not only increased more than 10 times the expression of IFN response genes but also induced demethylation of the apoptosis-associated IFN response gene *XAF1* promoter. Interestingly, MG98 also defeated the resistance to IFN-induced apoptosis. Either 5-aza-2′-deoxycytidine or MG98 depleted DNMT1 leading to reactivation of cancer-testis antigens MAGE and RAGE in ACHN cells which might be relevant for immune modulation (Reu et al., 2006). In a pre-clinical assay, exposure of RCC cell lines (Caki-1, 786-O, and A498) to 5-aza-2′-deoxycytidine enhanced the cytotoxicity of vinblastine (VBL), a classical cytotoxic drug against RCC. 5-aza-2′-deoxycytidine led to demethylation and re-expression of *connexin 32* (*Cx32*) which directly contributed to downregulation of P-gp by activation of c-Jun NH_2_-terminal kinase (JNK). These results suggest that re-expression of *Cx32* increases RCC cells response to VBL (Takano et al., 2010). Notably, the co-treatment of Caki-1 xenograft mice with 5-aza-2′-deoxycytidine and VBL led not only to suppression of tumor volume and weight but also reduced the expression of *P-gp, Bcl-2* and *cyclin B1*. This combined effect appear to be mediated by the accumulation of intracellular VBL and by apoptosis and cell cycle arrest induction (Iwata et al., 2011). Likewise, combination of 5-aza-2′-deoxycytidine with paclitaxel (PTX) synergistically inhibited RCC cell growth. Both drugs suppressed RCC cell proliferation by inducing G_2_/M cell cycle arrest and PTX also enhanced tumor cell apoptosis in a dose-dependent manner. Thus, this synergistic growth suppression of RCC cells suggests that this DNMTi could remarkably increase the susceptibility of RCC to PTX (Shang et al., 2007).

#### Clinical trials

Monotherapy with 5-aza-2′-deoxycytidine was scheduled in a phase I study at doses from 2.5 to 20 mg/m^2^ on days 1–5 in 31 patients with refractory malignancies, including three RCC subjects. Although 5-aza-2′-deoxycytidine decreased DNA methylation both in tumor and in peripheral blood mononuclear cells (PBMCs), there was no correlation between these two parameters. However, this agent induced apoptosis and increased CTR1 (copper transporter) expression through methylation-independent mechanisms (Stewart et al., 2009). A phase I clinical trial that combined 5-aza-2′-deoxycytidine, daily subcutaneously injected during 5 days at weeks 1 and 2 of a 12-week cycle, with high-dose interleukin-2 (600,000 IU/Kg), administered intravenously 14 times every 8 h, resulted in stable disease in 3 of the 5 RCC patients enrolled. Grade 4 neutropenia was observed (Gollob et al., 2006).

Another phase I trial, that enrolled 55 patients with advanced disease from which two were RCC patients, combined 5-azacytidine subcutaneously administered with oral valproic acid. One RCC patient presented stable disease for 6 months with a significant increase in histone acetylation. Grade 1 and 2 toxicities were reported (Braiteh et al., 2008).

Finally, in a phase I/II trial (NCT00003890) that enrolled 20 metastatic renal carcinoma patients, the anti-tumor activity of MG98 was assessed. This compound was intravenously administered at a dose of 360 mg/m^2^ twice weekly for three consecutive weeks out of four. The most common symptomatic toxicities were rigors, fatigue, fever, and nausea. Unfortunately, the results did not show a conclusive pattern of decreased DNMT1 activity in PBMCs post MG98 treatment (Winquist et al., 2006). However, a recent clinical study with advanced RCC patients demonstrated a great tolerance for combination of MG98 and IFN-α-2β in an intermittent schedule rather than continuous. Indeed, one patient showed a partial response, one had symptomatic improvements and eight patients achieved stable disease after combined intermittent treatment (Amato et al., 2012).

### Prostate cancer

PCa is the second most commonly diagnosed cancer and the 5th leading cause of cancer related death in men worldwide (Ferlay et al., 2014).

When organ-confined, PCa is curable by radical prostatectomy and/or radiation therapy (Kohli and Tindall, 2010). The use of serum PSA for PCa detection lacks sensitivity and specificity, leading to a relatively high frequency of unnecessary prostate biopsies, which is an expensive and invasive procedure (Catalona et al., 2000). Consequently, a substantial proportion of patients with indolent tumors is overdiagnosed and overtreated, from which several might experience side-effects, such as urinary incontinence and erectile dysfunction, without significant clinical benefit (Troyer et al., 2004). For advanced disease, the treatment of choice is androgen deprivation therapy (ADT) which aims to reduce the levels of male hormones. Although most patients initially respond to this therapy, after 18–30 months, approximately 30% of the cases progress to the lethal stage of this disease, designated castration-resistant PCa (CRPC) (Heidenreich et al., 2014). Despite treatment with secondary hormonal therapeutic agents, such as abiraterone acetate and enzalutamide, acquired resistance inevitably occurs after a few months (Watson et al., 2015). For metastatic CRPC (mCRPC), chemotherapy with docetaxel represents the standard therapy, however, the median time to progression remains 6–8 months and overall survival remains less than 2 years (Petrylak et al., 2004; Tannock et al., 2004). Mitoxantrone alone or in combination with prednisone, the radiopharmaceutical radium-223 and the autologous cellular immunotherapy Sipuleucel T may also be used for mCRPC with a significant, although limited survival benefit (Berthold et al., 2008). Unfortunately, none of these agents are curative, reinforcing the urgent need for investigation of new therapeutic strategies.

Epigenetic changes, especially aberrant DNA methylation, play an important role in PCa development and progression (Perry et al., 2006; Schulz and Hatina, 2006). Among all solid malignancies assessed by TCGA consortium, PCa is the one that exhibits the lowest *DNMT3A/3B* expression levels (Cerami et al., 2012; Gao et al., 2013). In PCa, promoter hypermethylation is directly involved in silencing of several TSGs, such as *APC, GSTP1, RAR*β*2, RASSF1A*, estrogen genes, cell adhesion genes (*CD44* and *CDH1*), cell cycle control genes (*CCND2, CDKN1B*, and *SFN*), apoptotic genes (*PYCARD, RPRM*, and *GLIR1*), and also androgen receptor (*AR*) which is involved in progression to CRPC (Jerónimo et al., 2001, 2004; Maruyama et al., 2002; Sasaki et al., 2002; Kang et al., 2004; Yegnasubramanian et al., 2004; Henrique et al., 2007; Carvalho J. R. et al., 2010). Methylation of the *RASSF1A* gene promoter was strongly correlated with increased risk of recurrence of PCa, aggressiveness and tumor progression (Liu et al., 2002). Importantly, progression to CRPC has also been linked to *AR* silencing by hypermethylation which was in fact, described in 30% of the CRPC (Suzuki et al., 2003; Schayek et al., 2010). *GSTP1* aberrant hypermethylation is one of the most frequent alterations in PCa, being present in over 90% of tumors and even in 75% of prostatic intraepithelial neoplasia, a PCa precursor lesion (Brooks et al., 1998; Kang et al., 2004). Therefore it constitutes the most promising epigenetic biomarker for detection of this malignant neoplasm (Henrique and Jerónimo, 2004). Global hypomethylation occurs either in primary PCa or, more extensively, in metastatic disease (Yegnasubramanian et al., 2008). Increased tumor grade (Gleason score ≥ 7) has been associated not only with widespread genome hypomethylation but also with promoter hypermethylation of several individual loci, including *ABCB1, APC, GSTP1, PTGS2, PYCARD, RAR*β*2*, and *RASSFA1* (Florl et al., 2004; Kang et al., 2004).

#### Pre-clinical studies

Numerous pre-clinical assays assessed the usefulness of DNMTi for PCa treatment. Gravina and co-workers, showed that chronic exposure (20 days) of PCa cell lines to 5-azacytidine, resulted in a significant decrease of tumor cell proliferation and increase in AR and PSA protein levels. Furthermore, PCa cell lines increased sensitivity to the apoptotic effects of bicalutamide, an anti-androgen used in the clinics (Gravina et al., 2008), and restoration of *AR* also sensitized xenograft models of CRPC to this anti-androgen (Gravina et al., 2010).

PCa cells exposure to 5-aza-2′-deoxycitidine increased the level of *plasmonigen activator inhibitor-1* (*PAI-1*) transcript and restored the pro-inflammatory cytokines effects (Hagelgans et al., 2013). Moreover, 5-aza-2′-deoxycitidine led to a significant suppression of cell proliferation, induction of cell death, and demethylation of *GSTP1* promoter, with associated protein re-expression (Chiam et al., 2011). Recently, exposure of PCa cell lines to 5-aza-2′-deoxycytidine resulted in the re-expression of *KAI1*, a metastasis suppressor gene, found hypermethylated in PCa (Lee et al., 2016). 5-aza-2′-deoxycitidine was able to decrease PCa cell stemness and induce a more differentiated status. *in vitro* and *in vivo* assays demonstrated that *AR* re-expression associated with the reversion of its methylation pattern led to suppression of PCa stem cells self-renewal, with a consequent decrease in tumorigenesis (Tian et al., 2012). Restoration of *ASC/TMS1* expression in LNCaP cells was only achieved with 5-aza-2′-deoxycytidine while TSA did not increase gene expression (Das et al., 2006). Exposure to 5-aza-2′-deoxycytidine also prevented cancer development in TRAMP mice, reduced the formation of lymph node metastases and significantly increased survival (McCabe et al., 2006).

Zebularine not only requires a 100-fold higher dose than 5-aza-2′-deoxycitidine to inhibit cell proliferation but it is also less potent in inducing cell death, and fails to restore GST-pi protein expression (Chiam et al., 2011). On the other hand, zebularine restored sensitivity of PCa cells to the DNA minor groove binder brostallicin, which correlated with re-expression of two glutathione-S-transferase (GST)-detoxifying enzymes (GST-pi and GST-mu), *in vitro* and *in vivo* (Sabatino et al., 2013).

Regarding RG108, it induced dose and time dependent growth inhibition and apoptosis of PCa cell lines. This compound repressed DNMT activity and expression and reduced global DNA methylation of androgen-responsive PCa cell lines. Furthermore, chronic treatment (14 days) with RG108 significantly decreased promoter methylation levels of *GSTP1, APC*, and *RAR*β*2*, although mRNA re-expression was only succeeded for *GSTP1* and *APC* (Graça et al., 2014a).

Procainamide exposure led to reversion of *GSTP1* hypermethylation with concomitant restoration of gene expression not only in LNCaP cells but also in xenograft tumors of athymic nude mice (Lin et al., 2001).

Exposure of PCa cells to hydralazine led to cell growth and invasion inhibition, as well as apoptosis induction. Moreover, this compound also induced cell cycle arrest and DNA damage. Additionally, the exposure of PCa cells to hydralazine decreased *DNMT1, DNMT3A*, and *DNMT3B* mRNA levels as well as DNMT1 protein levels, which might be linked to the significant decrease in *GSTP1, BCL2*, and *CCND2* promoter methylation levels and concomitant transcript re-expression. Importantly, hydralazine restored AR expression, with upregulation of its target *p21* in DU145 cell line. The attenuated malignant phenotype of PCa cells was also associated with EGFR signaling pathway disruption (Graça et al., 2014b). One study comparing hydralazine and procainamide with 5-aza-2′-deoxycitidine demonstrated that 5-aza-2′-deoxycitidine was considerably more effective in demethylating and reactivating TSGs in PCa cell lines than non-nucleoside analog inhibitors (Chuang et al., 2005).

SGI-1027 also depleted DNMT1 expression in LNCaP cells (Datta et al., 2009). Additionally, this compound and two analogs (paralmeta and metalmeta) were able to inhibit cell proliferation and viability of PC-3 cell line (Valente et al., 2014).

Disulfiram demonstrated a dose-dependent inhibition of DNMT1 activity, promoted PCa cells apoptosis and cell cycle arrest, and reduced global 5 mC content. This compound also led to re-expression of *APC* and *RAR*β*2* (Lin J. et al., 2011). Furthermore, it inhibited PCa cell proliferation, promoted cell-cycle arrest and apoptosis by re-expressing tumor suppressor *estrogen receptor-*β (*ER-*β) that is epigenetically silenced in PCa by promoter hypermethylation. This re-expression was mainly due to inhibition of total DNMT activity (Sharma et al., 2015).

Gemcitabine functionally inhibited DNA methyltransferase activity in both nuclear extract and recombinant protein, reactivating several epigenetically silenced genes, including *GSTP1, IGFBP3*, and *RASSF1A*. This compound also destabilized DNMT1 in PCa cell lines (LNCaP, 22Rv1 and DU145). Moreover, it demonstrated a similar activity to 5-aza-2'-deoxycitidine but at significantly lower concentrations compared to those achieved in the treatment of patients with solid tumors (Gray et al., 2012).

In a previous study aimed at discovering novel DNMT inhibitors, 1120 small compounds from a random collection were tested and the 12 most potent hits were selected for cytotoxicity tests in DU145 cell line. Notably, most of the compounds revealed inhibitory activity and cytotoxicity at low micromolar concentrations (Ceccaldi et al., 2013). Furthermore, these concentrations were able to inhibit DNMT, MBP, and HDAC enzymatic activity, reactivate methylated-silenced TSGs, induce histone acetylation and alter nucleosome positioning in PCa cell lines (Fang et al., 2007; Wang et al., 2007; Pandey and Gupta, 2009; Majid et al., 2010). Mahanine, a plant-derived carbazole alkaloid, restored *RASSF1A* expression in LNCaP and PC-3 cell lines possibly due to downregulation of DNMT1 and DNMT3B enzymes activity by inactivation of AKT pathway (Jagadeesh et al., 2007; Agarwal et al., 2013). In the same cell lines, genistein not only reversed GSTP1, RARβ2, and RASSF1A promoter methylation but also induced gene reactivation and respective protein expression (Fang et al., 2005; Vardi et al., 2010). Similarly, this compound induced DNMTs' downregulation in PCa cells, reducing *ER-*β promoter methylation with concomitant increased expression (Mahmoud et al., 2015). PEITC reduced DNMT protein levels and reactivated RASSF1A expression in LNCaP cells. Moreover this compound at 5 μM promoted early apoptosis and G2/M cell cycle arrest (Boyanapalli et al., 2016). TRAMP C1 cells exposure to curcumin resulted in *Nrf2* promoter methylation status reversion, a master regulator of the cellular antioxidant defense system, with concomitant gene re-expression (Khor et al., 2011). In addition, 5 μM of curcumin reversed CpG methylation of *Neurog1* promoter region, a cancer methylation marker usually hypermethylated in PCa and whose expression is also altered in LNCaP cells (Shu et al., 2011).

### Combined therapy

One of the major obstacles for CRPC treatment is the development of resistance to therapy. Taking this into account, a pre-clinical assay evaluated the synergistic effect of 5-azacytidine in association with docetaxel and cisplatin in aggressive PCa models. The results demonstrated a significant reduction of tumor cell proliferation, induction of apoptosis and sensitization of xenografts to docetaxel and cisplatin treatments (Festuccia et al., 2009).

Promoter hypermethylation of *ASC*, which causes gene silencing and associates with biochemical recurrence and aggressive disease, was reversed after exposure to 5-aza-2′-deoxycytidine and zebularine, in five PCa cell lines (Collard et al., 2006). Fialova et al. combined 5-aza-2′-deoxycitidine with a histone deacetylase inhibitor (sodium butyrate, NaB) and found a significant decrease of cell viability, as well as site-specific demethylation at the *AR* promoter region followed by gene re-expression and increased acetylation of histones H3 and H4 (Fialova et al., 2013). When combined with the chemotherapeutic agent paclitaxel (PTX), 5-aza-2′-deoxycitidine enhanced the apoptotic effects and the arrest at G_2_/M cell cycle phase of this drug. This treatment strategy achieved synergistic growth suppression in all PCa cell lines, and could be an alternative for clinical management of this disease (Shang et al., 2009). Likewise, the combination of cisplatinum with 5-aza-2′-deoxycitidine resulted in a great synergy in triggering apoptotic death of DU145 cells (Fang et al., 2004). Recently, a novel 5-aza-2′-deoxycitidine formulation based on the use of engineered erythrocyte (Erythro-Magneto-Hemmagglutinin Virosomes, EMHVs) drug delivery system (DDS) which aims to reduce the incidence of toxicity on healthy tissues was described. This novel magnetic EMHV DDS improved the stability of the carried drug and exhibited high efficiency in confining its delivery at the site of action, *in vivo*. Moreover, it induced a significant tumor mass reduction in PCa xenografts models at a concentration which is 700-fold lower than the normal therapeutic dose. This innovative approach might be attractive for treatment of solid tumors (Naldi et al., 2014).

A recent study reported that the combination of genistein with daidzein, two soy isoflavones, resulted in a synergistic effect, inhibiting cell proliferation and inducing apoptosis of PCa cells (Dong et al., 2013).

The combination of PEITC with curcumin, induced cellular growth arrest and apoptosis more effectively than either compound alone, through inhibition of protein kinase B and NFkB pathways, in PCa cell lines and PC-3 xenografts (Khor et al., 2006).

#### Clinical trials

A phase I/II clinical trial (NCT00503984) evaluated the combination of 5-azacytidine, docetaxel, and prednisone in patients with mCRPC whose disease had progressed during or after docetaxel therapy. In phase I, 5-azacytidine and docetaxel were alternately escalated with 75 mg/m^2^ each in a standard 3+3 design. In phase II, all patients received 5-azacytidine 150 mg/m^2^ daily for 5 days followed by docetaxel 75 mg/m^2^ on day 6, every 21 days, and continuous prednisone 5 mg twice daily. A significant reduction in GADD45 methylation was observed after treatment with 5-azacytidine. This phase I/II trial showed that the combination of 5-azacytidine, docetaxel and prednisone with growth factor support is an option for mCRPC patients providing a median progression free survival of 4.9 months for all patients and a median overall survival of 19.5 months for 4 of the 22 patients (18 deaths) (Singal et al., 2015). An open label phase II study (NCT00384839) that enrolled 36 patients, evaluated 5-azacytidine effects in men with progressive metastatic or non-metastatic CRPC in combined androgen blockade (CAB) with a PSA-DT < 3 months. The primary endpoint of PSA-DT ≥ 3 months during any cycle was achieved in 19 patients. Eleven patients experienced at least one PSA-DT ≥ 6 months and nine experienced at least one PSA-DT ≥ 9 months. Twenty-four patients (70.6%) demonstrated some deceleration of PSA-DT during any cycle of treatment compared with baseline, and the median overall PSA-DT during the entire duration of therapy was 2.8 months compared with the baseline of 1.5 months. The median clinical progression-free survival for all 36 patients was 12.4 weeks. Grade 3 toxicities included fatigue and neutropenia with 4 patients discontinuing the treatment due to toxicity (Sonpavde et al., 2011). Another phase I trial that combined 5-azacytidine and valproic acid enrolled 55 patients with advanced malignancies, two of them with PCa. A significant decrease in global DNA methylation and induction of histone acetylation with stable disease lasting 6 months in PCa patients were achieved. Neutropenic fever and thrombocytopenia were identified as dose-limiting toxicities (Braiteh et al., 2008).

Thibault and co-workers, conducted a phase II study with 5-aza-2′-deoxycitidine at 75 mg/m^2^ in 14 men with progressive, metastatic PCa recurrent after total androgen blockade and flutamide withdrawal. Two of the 12 patients evaluable had stable disease with a time to progression of more than 10 weeks. These results disclose that 5-aza-2′-deoxycitidine is a well-tolerated regime with modest clinical activity against CRPC (Thibault et al., 1998).

A pilot clinical trial (NCT01917890) assessed the efficacy of curcumin supplementation in 40 PCa patients treated with external beam radiotherapy. Patients were divided into two groups with a random selection of those which received 3 g/day curcumin orally (*n* = 20) and a placebo group (*n* = 20). Interestingly, patients that received the curcumin regime presented less urinary symptoms, suggesting that this natural compound could offer some radioprotective effect (Hejazi et al., 2013).

In a dose escalation phase I trial (NCT01118741) with disulfiram in 19 men non-metastatic recurrent PCa after local therapy, five patients of two different cohorts achieved a transient global demethylation response. However, disulfiram was poorly tolerated in six patients, who experienced grade 3 toxicities (Schweizer et al., 2013).

### Testicular cancer

Although, testicular cancer is relatively rare, it is the most common solid tumor in young Caucasian men aged 15–39 years old (Ferlay et al., 2014). Testicular germ cell tumors (TGCTs) represent more than 95% of all testicular cancers and are thought to derive from primordial germ cells or early gonocytes (Houldsworth et al., 2006; Clark, 2007). Generally, they are classified into two distinctive histological subtypes: seminomas (40%) and non-seminomas (60%), which share the same precursor lesion, testicular intraepithelial neoplasia/germ cell neoplasia in situ (Oosterhuis and Looijenga, 2005). TGCTs patients are treated with orchiectomy and, subsequently, with radiotherapy and/or chemotherapy, depending on the histology and clinical stage (Oosterhuis and Looijenga, 2005). Even patients with metastatic disease are often successfully treated with cisplatin-based chemotherapy, displaying 5-year survival rates over 70% (Giuliano et al., 2006). However, 15–20% of the patients are refractory to this treatment and about 15% present a later relapse and develop progressive disease (Horwich et al., 2006; Koychev et al., 2011; Hanna and Einhorn, 2014). Unfortunately, there are no effective therapies for these patients.

In general, TGCTs display global DNA hypomethylation (Netto et al., 2008; Wermann et al., 2010) and exhibit the highest expression of *DNMT3A/3B* amongst solid tumors (Cerami et al., 2012; Gao et al., 2013). Seminomas are largely unmethylated, while nonseminomas have a global methylation status that differ according to their degree of differentiation (Smiraglia et al., 2002; Wermann et al., 2010). Nonseminomas present undifferentiated and pluripotent cells, known as embryonal carcinoma (EC) cells that are proposed to be TGCTs stem cells and the malignant homologous of embryonic stem cells (Houldsworth et al., 2006; Clark, 2007; Kristensen et al., 2008). Several promoters have been reported to be methylated in TGCTs, including *APC, ARF, BRCA1, CALCA, CCNA1, HOXA9, MGMT, hMLH1, PRSS21, RAR*β*2, RASSF1A, SCGB3A*, and *TP53* (Lind et al., 2006; Taberlay and Jones, 2011). Importantly, *RASSF1A* and *HIC1* hypermethylation was previously associated with cisplatin resistance in EC cell lines (Koul et al., 2004). Conversely, it was shown that *LINE1* is extremely hypomethylated in both TGCTs subtypes (Ushida et al., 2012). A recent report described that an elevated methylation frequency of *CALCA* and *MGMT* was present in nonseminomas and was related with poor clinical outcome in TGCTs patients. In addition, *CALCA* hypermethylation was associated with refractory disease (da Silva Martinelli et al., 2016).

#### Pre-clinical studies

5-aza-2′-deoxycytidine was able to induce apoptosis of human teratocarcinoma stem cells, but not in differentiated cells derived from human nullipotent EC cells. Intriguingly, expression of DNMT3B is required for induction of apoptosis and differentiation of human teratocarcinoma stem cells by 5-aza-2′-deoxycytidine (Wongtrakoongate et al., 2014). Exposure of NT2/D1 cells to low concentrations of 5-aza-2′-deoxycitidine resulted in DNA damage, induction of *p53*, as well as global and gene specific promoter DNA demethylation (*RIN1, SOX15, GPER*, and *TLR4*). Additionally, this treatment also led to downregulation of genes associated with pluripotency, like *NANOG, SOX2, GDF3*, and *Myc* target genes (Biswal et al., 2012).

Intriguingly, low concentrations of SGI-110 decreased tumor cell growth not only of cisplatin sensitive EC cells NT2/D1 and cisplatin resistant NT2/D1-R1, but also in a xenograft model of cisplatin resistant TGCT. Importantly, this compound re-sensitized refractory EC cells to cisplatin both *in vitro* and *in vivo* models. The expression of *GDF15, CDKN1A*, and *GADD45A* (p53 target genes), *RASSF1* and *SOX15* was induced after SGI-110 exposure. Moreover, in xenograft models, SGI-110 increased the expression of immune pathway genes (Albany et al., 2017).

#### Combined therapy

Exposure of human EC cells to low nanomolar concentrations of 5-aza-2′-deoxycitidine resulted in a significant decrease of cell proliferation and survival. This phenotype was associated with ATM pathway activation, H2AX phosphorylation, p21 increased expression and induction of genes known to be methylated in TGCTs like *MGMT, RASSF1A* and *HOXA9*. Notably, not only cisplatin-resistant EC cells retained sensitivity to low concentrations of 5-aza-2′-deoxycytidine but also pretreatment with this agent re-sensitized those cells to cisplatin-mediated toxicity. Moreover, knockdown of *DNMT3B* in EC cells reduced cell sensitivity to 5-aza-2′-deoxycytidine, supporting the role of *DNMT3B* in 5-aza-2′-deoxycytidine treatment response (Beyrouthy et al., 2009).

A recent study with an intrinsically platinum-resistant seminoma cell line (TCam-2) disclosed that 5-azacytidine treatment re-sensitize these cells to cisplatin. Furthermore, after demethylation, the stem cell markers *NANOG* and *POU5F1*, as well as *VASA* (the germ cell-specific marker), displayed increased expression (Wermann et al., 2010).

#### Clinical trials

A phase II clinical trial dating from 1977, which enrolled 214 patients with solid cancers, evaluated the effect of high doses of 5-azacytidine. Of the four evaluable testicular cancer patients, two developed partial responses with 5-azacytidine doses from 225 to 150 mg/m^2^ (Quagliana et al., 1976). Roth and colleagues performed a phase II trial which enrolled 17 patients with germ cell tumors refractory to cisplatin treatment. Patients received 5-azacytidine at a dosage of 150 mg/m^2^/day at days 1 to 5 by continuous infusion every 3 weeks. All patients progressed on 5-azacytidine and grade 3 and 4 toxicities were observed being the most important granulocytopenia and anemia. Therefore, the authors were unable to confirm 5-azacytidine activity on patients with germ cell tumors (Roth et al., 1993).

A single-arm phase II study (NCT00404508) demonstrated that the combination of hydralazine with magnesium valproate before chemotherapy resulted in decreased chemotherapy resistance with stable clinical response in one patient with refractory non-seminomatous TGCT. Besides that, it was observed a clinical benefit concerning progression-free survival and overall survival of 5.6 months and 5.7 months, respectively (Candelaria et al., 2007).

## Discussion and conclusions

Urological cancers account for a sizeable proportion of cancer-related mortality and morbidity (Ferlay et al., 2010). Thus, discovery of novel therapeutic strategies, ideally based on tumor biology, are pivotal to change this scenario. Epigenetic alterations are a common feature of urological tumors and are implicated in its genesis (Esteller, 2008). One of the most widely studied epigenetic alterations in urological tumors is aberrant DNA methylation. Thus, effective targeting of the enzymes that catalyze DNA methylation—the DNMTs—constitutes a major research goal in this field. One of the major challenges regarding the use of epigenetic therapies is the establishment of a therapeutic window that may reduce treatment-related toxicity, mostly preserving normal tissues and selectively targeting tumor cells. Despite the successful results achieved in pre-clinical studies and contrarily to hematological malignancies, DNMTi monotherapy clinical trials in solid tumors have not shown significant anti-cancer efficacy, thus far (Issa and Kantarjian, 2009). The slower proliferative rate of solid cancers compared to hematolymphoid neoplasms as well as toxicity due to higher dosages might account for the negative results of nucleoside analogs. Remarkably, low DNMTi dose schedules over extended periods of time have been proven safe and effective in reprogramming cancer cells since cytotoxic effects were not immediately induced, such as DNA damage, cell cycle arrest and apoptosis. Indeed, transient exposure to low-dose DNMTi is associated with long-term anti-cancer “memory” therapy, which reduces self-renewal, and sustains decreased genome-wide promoter DNA methylation and gene re-expression, as well as anti-neoplastic changes in critical regulatory cellular pathways (Tsai et al., 2012). Furthermore, these agents exhibit less toxicity when compared to conventional chemotherapy. Non-nucleoside analogs, on the other hand, display reduced efficacy compared to nucleoside analogs, notwithstanding their safer clinical profile (Brueckner et al., 2007). Hopefully, the combination of demethylating agents with HDAC inhibitors might prove beneficial, not only due to their synergistic action leading to TSGs re-expression in cancer cells, but also because both agents demonstrate angiostatic effects (Braiteh et al., 2008; Fialova et al., 2013).

The unsatisfactory clinical activity of most DNMTi in urological cancer patients might also be related with the short half-life of first-generation DNMTi. The combined administration of epigenetic-based therapy with other anti-cancer strategies, such as chemotherapy, radiotherapy, hormonal therapy and immunotherapy, might sensitize cancer cells and potentiate therapeutic effectiveness, materializing the “epigenetic priming” concept (ClinicalTrials.gov, 2007; Gravina et al., 2008; Hejazi et al., 2013; Singal et al., 2015). Indeed, epigenetic immunomodulation seems to more effectively prime and sensitize the host immune system to immunotherapies, by means of mechanisms such as the stimulation of viral defense pathways (Dunn and Rao, 2017). Interestingly, several studies have confirmed the efficacy of immunotherapy in BlCa and RCC patients, suggesting that the combination of DNMTi with immune checkpoint inhibitors might constitute a valid therapeutic strategy to be explored in the near future (Reu et al., 2006; ClinicalTrials.gov, 2016b; Massard et al., 2016). A possible explanation is that although DNA demethylating agents promote an increase of T cells at the tumor site, these T cells may be repressed by immune checkpoint molecules, i.e., CTLA-4 and PD-L1, leading to immune escape. However, the dependence on immune repression might increase tumor responsiveness to immune checkpoint inhibitors, such as anti-CTLA-4, anti-PD-L1, and anti-PD1 (Roulois et al., 2016). In fact, in a phase I/II clinical trial, non-small-cell carcinoma lung cancer patients pre-treated with 5-azacytidine showed an improved clinical response to subsequent anti-PD1 therapy (Juergens et al., 2011).

Concerning PCa, several trials are now focused in combining ADT or chemotherapy with epigenetic modulators to increase efficacy of those standard therapies, avoiding epigenetic reprograming and re-sensitizing hormone-refractory disease (Sonpavde et al., 2011; Singal et al., 2015). It is now accepted that epigenetic reprogramming is a major player in the development of therapy-evading tumor. Importantly, these approaches intend to extend long-term clinical responses in CRPC patients. For testicular cancer, only a few pre-clinical studies and clinical trials have been performed, and, thus, epigenetic-based therapy constitutes a largely unexplored field, especially the combined use of DNMTi with conventional chemotherapy.

The development of new agents for targeting cancer epigenome should also be considered and it includes new derivatives from currently used drugs, as well as novel drug design concepts. The discovery/development of new drugs designed to inhibit both DNMT3A and 3B, as well as DNMT1, might be of great clinical interest, in addition to increased understanding of their functions. Furthermore, translating pre-clinical epigenetic discoveries in the clinical setting has proven to be challenging, with the intrinsic complexity of human epigenetics as the most important one. Additionally, DNMTi currently in clinical trials display low selectivity to specific targets and, thus, it is difficult to predict their effects owing to the redundancy and crosstalk among the increasingly large set of epigenetic-modulating enzymes. Therefore, future studies should tackle this important hurdle. Three other issues should also be taken into consideration: (1) the same gene might play different roles in carcinogenesis in different tumors, (2) the same modification at a specific gene locus may have distinct implications on different tissues, and (3) within the same tumor model, different epigenetic alterations may drive different cancer subtypes. Consequently, the design and selection of DNMTi should be tailored to the heterogeneous biology of urological malignancies.

To effectively address the value of DNMTi as therapeutic agents, a molecular approach must also be considered. There is a need to disclose not only the hypermethylated gene profile specific to tumor onset and progression but also define which gene sets might predict therapeutic response or resistance to DNMTi. Recent advances in molecular technology, including next-generation techniques, are now providing a wealth of new information on DNA methylation, enabling a more comprehensive analysis. However, this might refute previously established “dogma” and may generate great controversy.

Despite the aforementioned drawbacks, “epi-drugs” already disclosed promising results with some clinical benefit, mainly as sensitizers for conventional therapies. Remarkably, the establishment of a global epigenetic approach might be useful for improved understanding of cancer pathology, founding the basis for new strategies and future development of new drugs that might improve cancer patients' survival and quality of life.

## Author contributions

ÂM-M and IG collected the information. ÂM-M, IG, RH, and CJ wrote and revised the paper. ÂM-M have drawn Figures 1, 2. ÂM-M and IG draw Figure 3. All authors read and approved the final manuscript.

### Conflict of interest statement

The authors declare that the research was conducted in the absence of any commercial or financial relationships that could be construed as a potential conflict of interest.
